# Mortality, Morbidity, and Developmental Outcomes in Infants Born to Women Who Received Either Mefloquine or Sulfadoxine-Pyrimethamine as Intermittent Preventive Treatment of Malaria in Pregnancy: A Cohort Study

**DOI:** 10.1371/journal.pmed.1001964

**Published:** 2016-02-23

**Authors:** María Rupérez, Raquel González, Ghyslain Mombo-Ngoma, Abdunoor M. Kabanywanyi, Esperança Sevene, Smaïla Ouédraogo, Mwaka A. Kakolwa, Anifa Vala, Manfred Accrombessi, Valérie Briand, John J. Aponte, Rella Manego Zoleko, Ayôla A. Adegnika, Michel Cot, Peter G. Kremsner, Achille Massougbodji, Salim Abdulla, Michael Ramharter, Eusébio Macete, Clara Menéndez

**Affiliations:** 1 ISGlobal, Barcelona Ctr. Int. Health Res. (CRESIB), Hospital Clínic - Universitat de Barcelona, Barcelona, Spain; 2 Manhiça Health Research Centre (CISM), Manhiça, Mozambique; 3 Consorcio de Investigación Biomédica en Red de Epidemiología y Salud Pública (CIBERESP), Barcelona, Spain; 4 Centre de Recherches Médicales de Lambaréné, Albert Schweitzer Hospital, Lambaréné, Gabon; 5 Institute of Tropical Medicine, University of Tübingen, Tübingen, Germany; 6 Department of Parasitology, Leiden Medical University Center, Leiden, The Netherlands; 7 Ngounié Medical Research Centre, Fougamou, Gabon; 8 Ifakara Health Institute (IHI), Dodoma, Tanzania; 9 Faculté des Sciences de la Santé (FSS), Université d’Abomey-Calavi, Cotonou, Benin; 10 Institut de Recherche pour le Développement (IRD), Paris, France; 11 Université René Descartes, Paris, France; 12 Division of Infectious Diseases and Tropical Medicine I, Department of Medicine I, Medical University of Vienna, Vienna, Austria; Mahidol-Oxford Tropical Medicine Research Unit, THAILAND

## Abstract

**Background:**

Little is known about the effects of intermittent preventive treatment of malaria in pregnancy (IPTp) on the health of sub-Saharan African infants. We have evaluated the safety of IPTp with mefloquine (MQ) compared to sulfadoxine-pyrimethamine (SP) for important infant health and developmental outcomes.

**Methods and Findings:**

In the context of a multicenter randomized controlled trial evaluating the safety and efficacy of IPTp with MQ compared to SP in pregnancy carried out in four sub-Saharan countries (Mozambique, Benin, Gabon, and Tanzania), 4,247 newborns, 2,815 born to women who received MQ and 1,432 born to women who received SP for IPTp, were followed up until 12 mo of age. Anthropometric parameters and psychomotor development were assessed at 1, 9, and 12 mo of age, and the incidence of malaria, anemia, hospital admissions, outpatient visits, and mortality were determined until 12 mo of age. No significant differences were found in the proportion of infants with stunting, underweight, wasting, and severe acute malnutrition at 1, 9, and 12 mo of age between infants born to women who were on IPTp with MQ versus SP. Except for three items evaluated at 9 mo of age, no significant differences were observed in the psychomotor development milestones assessed. Incidence of malaria, anemia, hospital admissions, outpatient visits, and mortality were similar between the two groups. Information on the outcomes at 12 mo of age was unavailable in 26% of the infants, 761 (27%) from the MQ group and 377 (26%) from the SP group. Reasons for not completing the study were death (4% of total study population), study withdrawal (6%), migration (8%), and loss to follow-up (9%).

**Conclusions:**

No significant differences were found between IPTp with MQ and SP administered in pregnancy on infant mortality, morbidity, and nutritional outcomes. The poorer performance on certain psychomotor development milestones at 9 mo of age in children born to women in the MQ group compared to those in the SP group may deserve further studies.

**Trial registration:**

ClinicalTrials.gov NCT00811421

## Abstract 2

In a cohort study, Clara Menéndez and colleagues evaluate mortality, morbidity, and developmental outcomes in infants born to women who were enrolled in a randomized controlled trial of intermittent preventive treatment of malaria in pregnancy

## Editors' Summary

**Background:**

Malaria is a mosquito-borne parasitic disease that kills more than 600,000 people every year. Most of these deaths occur among young children living in sub-Saharan Africa, but pregnant women living in Africa and their unborn children are also very vulnerable to malaria. Infection with malaria during pregnancy can cause severe maternal anemia (reduced red blood cell numbers), stillbirths, and pre-term and low-birth-weight babies, and is responsible for the deaths of many women and their babies. To prevent this loss of life, the World Health Organization (WHO) recommends that pregnant women living in areas of moderate to high transmission in Africa receive the antimalarial drug sulfadoxine-pyrimethamine (SP) at each scheduled antenatal visit after the first trimester (intermittent preventive treatment of malaria in pregnancy [IPTp]). In addition, WHO advises pregnant women to sleep under insecticide-treated bed nets to protect themselves from the bites of infected mosquitoes and recommends effective case management of pregnant women with malarial illness.

**Why Was This Study Done?:**

Because malaria parasites are becoming resistant to SP, other antimalarial drugs are being investigated for use in IPTp. A recent randomized controlled trial (a study that compares outcomes in participants assigned at random to receive different interventions) compared the efficacy and safety of IPTp with SP and with mefloquine (MQ, an antimalarial drug that, like SP, is considered safe throughout pregnancy) in pregnant women living in four countries in sub-Saharan Africa. In the trial, the women taking MQ had fewer episodes of clinical malaria than the women taking SP, but the tolerability of MQ was poorer than that of SP. Importantly, the prevalence of stillbirth and of low-birth-weight babies was similar in both trial arms (the prevalence of a condition is the proportion of the population with that condition). Here, by following the babies born to the women participating in this trial for up to 12 months, the researchers evaluate the longer term effects of IPTp with SP and MQ during pregnancy on infant health and development.

**What Did the Researchers Do and Find?:**

The researchers followed 2,815 babies born to women who received MQ for IPTp and 1,432 babies born to women who received SP for IPTp for up to 12 months after birth. They measured the babies’ weight and length at 1, 9, and 12 months, assessed the babies’ psychomotor development (the development of language, hearing and social skills, and motor skills such as limb movement and grasping) at the same ages, and recorded the incidence of malaria, anemia, hospital admissions, outpatient visits, and death among the babies during the first year of life. The researchers found no significant differences in the nutritional outcomes of the infants born to women who received MQ compared to the infants born to women who received SP (significant differences are differences in outcomes that are unlikely to have arisen by chance). There were also no significant differences in psychomotor development between the two groups of infants apart from an increased risk of being unable to stand without help, walk without support, or bring food to the mouth at 9 months among the children born to women who received MQ. Finally, the incidence of malaria, anemia, hospital admissions, outpatient visits, and death was similar in the infants born to the two groups of women.

**What Do These Findings Mean?:**

Information on the outcomes was not available for 26% of the babies at 12 months, which may limit the accuracy of these findings. Nevertheless, these findings suggest that IPTp with MQ was not associated with any increased risk of undernutrition, illness, or death among infants compared to IPTp with SP. The poorer performance on three psychomotor development milestones among the children born to women given MQ may be due to multiple testing rather than to true differences between the groups of infants—as more outcomes are investigated in a randomized trial, it becomes more likely that the intervention groups will differ on at least one outcome by chance. It might therefore be worth investigating psychomotor development further in children whose mothers have been exposed to MQ during pregnancy. Although the lower tolerability of MQ compared to SP for pregnant women in this trial that was reported previously means that MQ is unlikely to be used for IPTp, these findings are important because antimalarial regimens containing MQ are recommended for malaria treatment during pregnancy and MQ alone is recommended for malaria prevention in pregnant women traveling to countries affected by malaria.

**Additional Information:**

This list of resources contains links that can be accessed when viewing the PDF on a device or via the online version of the article at http://dx.doi.org/10.1371/journal.pmed.1001964.

- Two *PLOS Medicine* Research Articles by Gonzalez et al. describe the randomized controlled trial of the efficacy and safety of IPTp with MQ versus SP in HIV-negative women and in HIV-infected women; additional information about the trial is available from ClinicalTrials.gov and the Pan African Clinical Trials Registry
- Information is available from the World Health Organization on malaria (in several languages) and on malaria in pregnancy; information on IPTp and the current WHO policy recommendation on IPTp with SP are available; the *World Malaria Report 2015* provides details of the current global malaria situation, including information on malaria in individual African countries
- The US Centers for Disease Control and Prevention also provides information on malaria and recommendations for the use of MQ during pregnancy; a personal story about malaria in pregnancy is available
- Information is available from the Roll Back Malaria Partnership on all aspects of global malaria control
- The Malaria in Pregnancy Consortium is undertaking research into the prevention and treatment of malaria in pregnancy
- MedlinePlus provides links to additional information on malaria (in English and Spanish)

## Introduction

Malaria infection in pregnancy is a significant public health problem in endemic regions, especially in sub-Saharan Africa, where there are nearly 30 million pregnancies at risk of infection every year [1]. Maternal infection is an important contributor to anemia and low birth weight (LBW), as well as to overall morbidity and mortality in both the mother and the infant [2–15]. To prevent maternal infection in malaria endemic areas in Africa, WHO recommends the use of insecticide-treated bed nets and the administration of intermittent preventive treatment of malaria in pregnancy (IPTp) with sulfadoxine-pyrimethamine (SP) [16]. Prevention of malaria with IPTp with SP has been shown to reduce LBW deliveries, neonatal mortality, and maternal morbidity [5,6,11,12,16–18]. However, the spread of SP parasite resistance in sub-Saharan Africa has raised concerns regarding its long-term use for IPTp, and alternative drugs are being evaluated.

Despite the emergence of resistance to mefloquine (MQ) in parts of Southeast Asia, MQ retains high antimalarial activity in Africa and has been shown to be effective both for malaria treatment in pregnancy and for preventing malaria infection in all trimesters [19–21]. Concerns that have been raised about its safety in pregnancy have not been confirmed, and MQ is among the very few antimalarials considered safe throughout pregnancy and is recommended by WHO and the US Centers for Disease Control and Prevention for chemoprophylaxis in non-immune pregnant women of all gestational ages traveling to malaria endemic regions [22,23]. It has also been recently reclassified as pregnancy category B by the US Food and Drug Administration and was proved to be safe in pregnancy in a recent systematic review [24,25].

It is recommended that clinical research involving pregnant women include a plan to monitor the outcome of the pregnancy as well as the short- and long-term health status of the child [26]; however, follow-up of children is rarely done in most African settings, mainly because of the difficulties of carrying out adequate monitoring. In the case of malaria, little is known about the effects of preventive interventions against malaria during pregnancy on the infant’s health. Only two trials in Southeast Asia have assessed the effect of MQ administration during pregnancy on the offspring’s health over the first month of life. In one study MQ was compared with placebo for malaria prophylaxis, and in the other trial MQ combined with artesunate (AS) was compared with quinine for treatment of malaria episodes. In neither study was an increased risk of adverse health, growth, or developmental outcomes in infants born to women receiving MQ in pregnancy reported [27,28].

We recently reported the results of a multicenter randomized controlled trial comparing MQ and SP for IPTp and evaluating the tolerability of two different MQ regimens in pregnant women in four sub-Saharan countries [29]. Women taking MQ were found to have fewer episodes of clinical malaria than SP recipients, and pregnancy outcomes and safety profiles were similar in both groups. However, drug tolerability was worse for MQ than for SP, even when splitting MQ doses over 2 d. Infants born to women participating in the trial were followed up until 12 mo of age. We report here the nutritional outcomes, psychomotor development, morbidity, and survival of infants born to women receiving either MQ or SP as IPTp.

## Methods

### Ethics Statement and Participants’ Safety

As previously described [29], the study protocol and informed consent forms were reviewed and approved by the ethics committee of the Hospital Clinic of Barcelona (Spain), the Comité Consultatif de Déontologie et d’Éthique of the Institut de Recherche pour le Développement (France), and all local regulatory authorities and national ethics review committees of each country participating in the study (S1 Table). The trial was conducted under the provisions of the Declaration of Helsinki and in accordance with good clinical practices guidelines set up by WHO and by the International Conference on Harmonization. An independent data safety monitoring board was created prior to the beginning of the trial and regularly reviewed and monitored the safety data collected. The trial was registered prior to the enrollment of the first participant in both ClinicalTrials.gov (NCT00811421) and the Pan African Clinical Trials Registry (PACTR2010020001429343).

### Study Area and Population

The study was conducted between September 2009 and January 2013 in four sub-Saharan countries: Benin (Allada, Sékou, and Attogon), Gabon (Lambaréné and Fougamou), Tanzania (Makole and Chamwino), and Mozambique (Manhiça).

### Study Design

Details of the study have been reported elsewhere [29]. A total of 4,749 pregnant women were enrolled into a randomized open-label three-arm trial to compare MQ with SP as IPTp for the prevention of the adverse effects of malaria during pregnancy and to compare the tolerability of two different MQ administration regimens in the context of long-lasting insecticide-treated bed net use. The three study arms were (1) IPTp with SP, (2) IPTp with MQ (15 mg/kg) given once as a full dose, and (3) IPTp with MQ (15 mg/kg) split over 2 d. The primary objective of the trial was to assess the safety and efficacy of the study drugs in pregnancy, independently of the dose regimen, as detailed in the study protocol (S1 Text). There were 4,247 live births born to the trial participants, 1,432 born to women in the SP group and 2,815 born to women in the MQ group; the infants were followed up until 12 mo of age. Assessment of the effect of the administration of MQ versus SP in the mother on the infant’s health and survival was also included as a trial objective.

### Study Procedures

#### Enrollment of pregnant women

All pregnant women attending a participating antenatal clinic for the first time were screened for eligibility to participate in the study. Inclusion criteria were the following: permanent residence in the study area; gestational age ≤ 28 weeks; negative HIV test at recruitment; absence of history of allergy to sulfa drugs or MQ; absence of history of severe renal, hepatic, psychiatric, or neurological disease; and absence of MQ or halofantrine treatment in the preceding 4 wk. Women who met the inclusion criteria and signed an informed consent form were randomized to the SP group and received standard IPTp (three tablets of the fixed combination therapy containing 500 mg of sulfadoxine and 25 mg of pyrimethamine; Malastop, Sterop) or to the MQ group and received 15 mg/kg of MQ (tablets of 250 mg of MQ base; Lariam, Roche). The number of tablets for MQ was calculated according to body weight. For women allocated to the MQ split-dose group, the 15-mg/kg dose was divided into two halves and administered over two consecutive days, with the second half dose administered either at the antenatal clinic or at home by study personnel.

#### Infant follow-up

After delivery, live births were given a study number different from that of the mother in order to be uniquely identified. Women were asked to bring their children to the study health facility when the babies were 1 mo of age or coinciding with the first Expanded Program on Immunization visit at 6 wk, and also at 9 and 12 mo of age. At each visit, the baby’s weight and length were recorded, and psychomotor development was evaluated following a simplified adapted protocol that included assessment of gross and fine motor skills, language, audition, and social skills [30]. Participants who did not attend scheduled study visits were visited at home and encouraged to attend the health facility for completion of study visits. Throughout the follow-up, infants reporting sick at the health facility were seen by study health personnel. Unscheduled outpatient visits and hospital admissions were recorded in standardized questionnaires. A capillary blood sample was taken for malaria parasitemia examination and hemoglobin determination in infants who presented with fever or a history of fever in the last 24 h or who appeared pale. All children who did not attend the visit at 12 mo of age were visited at home to assess the infant’s residence and health status.

### Laboratory Methods

Hemoglobin (Hb) was determined using mobile devices in capillary blood samples (HemoCue [Eurotrol] and Hemocontrol [EKF Diagnostics]). Thick and thin blood films were stained and read for *Plasmodium* species detection according to standard, quality-controlled procedures[31,32].

### Data Management, Statistical Methods, and Definitions

The quality of data recorded in the study source documents and case report forms was monitored regularly following good clinical practices principles by the trials’ clinical monitor before the data were sent to the centralized database in Manhiça, Mozambique. Data were double-entered using OpenClinica Enterprise software for clinical data management. The analysis compared infants born to women receiving either SP or MQ, independently of the MQ regimen. The analysis was done on the modified intention-to-treat (mITT) cohort that included all live births born to women who met the inclusion criteria and had data on the outcomes of the main trial. The mITT analyses were adjusted by country. To include seasonality in the adjusted analysis, the duration of women’s recruitment was divided into eight periods, and the interaction terms between period of recruitment and country were included in the model, which allows modeling of the effect of period in each country independently.

Baseline characteristics of newborns at delivery were described using standard statistics. Proportions were compared between mother’s IPTp groups using Fisher’s exact test. Adjustments for covariates and possible confounders were made using logistic regression and robust estimates of the covariance (Huber method) using the method proposed by Zou [33]. Comparisons of proportions are presented as a relative risk (RR) or a reduction in the RR (1 − RR × 100%) if the RR is lower than 1. Continuous variables were compared between groups and adjusted for covariates and possible confounders using ordinary least squares regression. Main outcomes (nutritional outcomes; psychomotor development; incidences of clinical malaria, anemia, outpatient visits, and hospital admissions; and mortality) were compared between all children born to MQ recipients and SP recipients. Results with *p* < 0.05 were considered statistically significant, without allowance for multiple testing. Variables were transformed to the logarithm scale if normality was thereby improved. Nutritional analysis was adjusted by birth weight. *z-*Scores were calculated for the four WHO-recommended anthropometric indices to assess nutrition in infants: weight for age (underweight, severe acute malnutrition), height for age (stunting), weight for height (wasting), and mid-upper arm circumference, according to WHO standard definitions [34,35]. Participants with missing data were not included in the analysis. Clinical malaria was defined as fever (≥37. 5°C) or history of fever in the past 24 h or signs suggestive of malaria, reported through direct questioning in scheduled visits and active reporting in unscheduled visits, confirmed by a positive blood smear. Anemia was defined as Hb < 125 g/l in cord blood for neonates and Hb < 110 g/l in peripheral blood in infants. LBW was defined as birth weight < 2,500 g, and preterm as gestational age < 37 wk at delivery. The following psychomotor development milestones were assessed at each age: at 1 mo—(i) movement of four extremities symmetrically, (ii) muscle tone, (iii) following of objects, (iv) response to sounds, and (v) response to smiles; at 9 mo—(i) ability to sit without leaning, (ii) crawling, (iii) standing without help, (iv) walking without support, (v) grasping small objects, (vi) palm grasping, (vii) moving objects from one hand to the other, (viii) turning at voice, (ix) ability to say a word, and (x) bringing solid food to his/her mouth; and at 12 mo—(i) walking, (ii) pincer grasping, (iii) understanding orders, (iv) ability to say some words, and (v) drinking from a cup. Congenital abnormalities, hospital admissions, and deaths were considered serious adverse events (SAEs) and were confirmed and reported in specific questionnaires by a study clinician and notified to the sponsor and the data safety monitoring board. Diagnoses for the SAE reporting were codified using Medical Dictionary for Regulatory Activities preferred term and system organ class coding system [36]. Incidences of clinical malaria, anemia, infant mortality, hospital admissions, and outpatient attendances were estimated as the number of episodes per person over the time at risk. Time at risk was defined as the time from the start of follow-up (day of birth) until the end of follow-up (visit at 12 mo) or withdrawal due to censoring or death, whichever occurred first. In order to avoid counting twice the same episode of clinical malaria, participants did not contribute to the denominator or numerator during an arbitrary period of 28 d after an event of clinical malaria was confirmed. For hospital admissions and outpatient attendances, a maximum of one episode per day was allowed. Infant mortality rate was defined as the number of deaths per 1,000 live births per year at risk. The total number of events was compared between groups using negative binomial regression to take into account a possible extra Poisson variation due to different frailty of the participants. The mortality comparison is expressed as a relative rate. Study completion was defined as attendance to visit 3 at 12 mo of age, independently of completing the other previous study visits. Data analysis was performed using Stata version 13 (Stata Corporation).

## Results

### Baseline Characteristics of Study Participants

**Fig 1:**
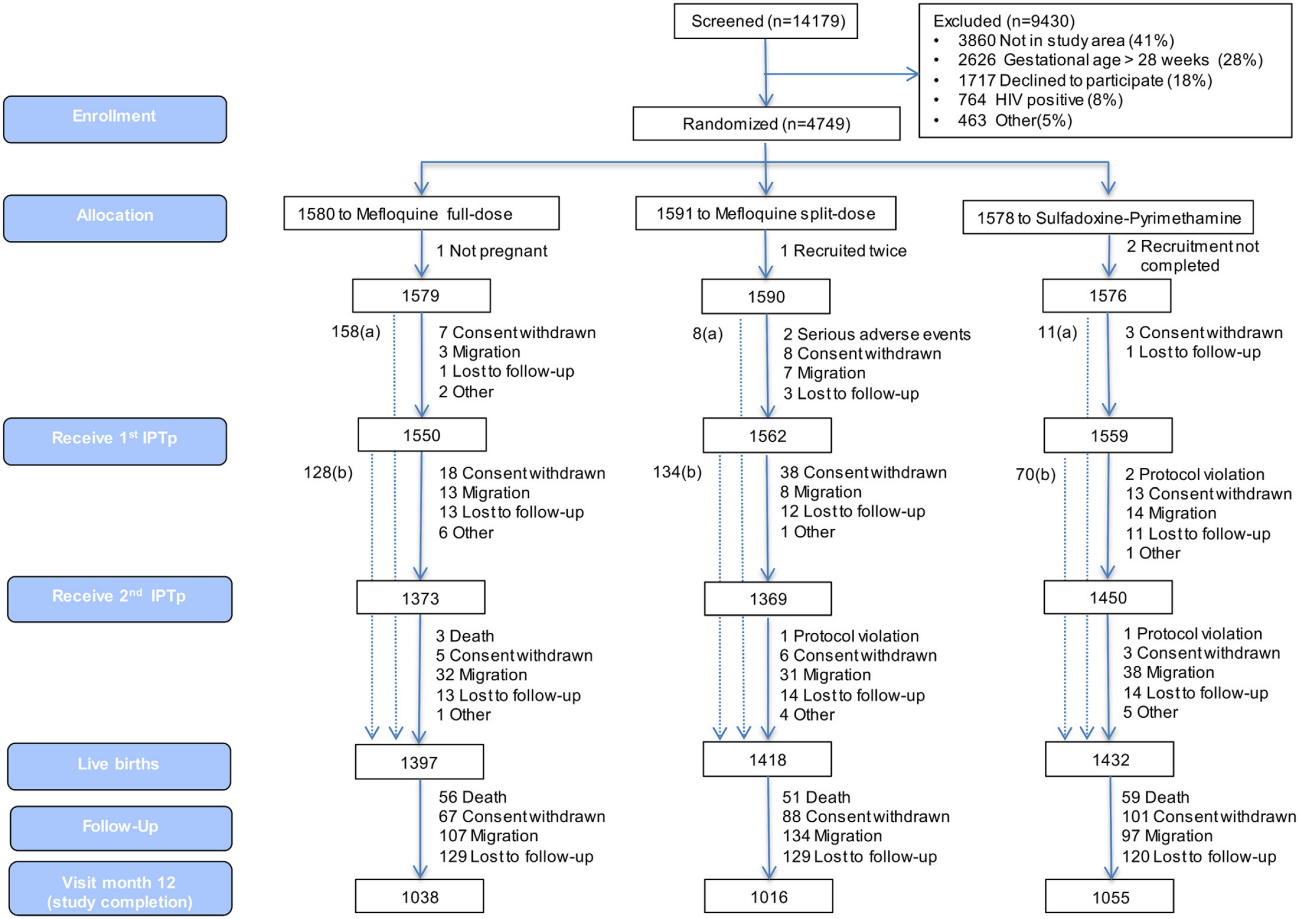
Trial profile (modified intention-to-treat cohort). ^a^Number of women recruited at first antenatal visit who did not attend the rest of the study visits but delivered at the maternity ward and for whom information on pregnancy outcome is available. ^b^Number of women who delivered before receiving second IPTp dose.

Fig 1 shows the trial profile of the mITT cohort that is the basis of this study. Overall, there were 2,815 and 1,432 live births born to mothers receiving IPTp with MQ and SP, respectively. The study follow-up was completed by 73% (2,054) of babies born to women allocated to the MQ group and by 74% (1,055) of those born to women in the SP group. Reasons for not completing the study were death (4% of total study population), withdrawal (6%), migration (8%), and loss to follow-up (9%). Infants who did not complete the study were similar at birth to those who did, except that they were in a lower proportion from Benin (17%), had a smaller mean head circumference (33.67 cm), a greater median gestational age (40 wk), and a lower proportion of cord blood anemia (8.8%) (S5 Table).

Baseline characteristics were similar for both study groups. Overall, mean prevalence of cord blood anemia, malaria parasitemia, and LBW was 10.6%, 0.3%, and 12.0%, respectively, with no significant differences between groups. Median gestational age at birth was 39 wk (interquartile range 38.4; 40.8), and congenital abnormalities were found in 46 (1.1%) live births (1.2% in the MQ group and 1.0% in the SP group); of these congenital abnormalities, 36 were detected at delivery, and ten during follow-up, with no significant differences between groups (Table 1).

**Table 1:** Baseline characteristics of study infants at delivery.

Characteristic	MQ Group		SP Group	
	*N*	Value	*N*	Value
**Live births by country**				
Benin	2,815	719 (25.5%)	1,432	370 (25.8%)
Gabon	2,815	663 (23.6%)	1,432	338 (23.6%)
Mozambique	2,815	736 (26.1%)	1,432	375 (26.2%)
Tanzania	2,815	697 (24.8%)	1,432	349 (24.4%)
**Male**	2,815	1,414 (50.2%)	1,432	718 (50.1%)
**Weight (g)**	2,791	3,016 (501)	1,423	3,017 (486)
**Length (cm)**	2,724	48 (4)	1,382	48 (5)
**Head circumference (cm)**	2,722	34 (2)	1,380	34 (2)
**Cord blood anemia** ^1^	2,815	304 (10.8%)	1,432	145 (10.1%)
**Cord blood parasitemia blood smear positive**	2,618	6 (0.2%)	1,324	4 (0.3%)
**Gestational age (wk)** ^2^ ^,^ ^3^	1,924	39.2 (38.4; 40.4)	980	39.6 (38.4; 40.8)
**Premature** ^2^ ^,^ ^3^ ^,^ ^4^	1,924	121 (6.3%)	980	56 (5.7%)
**LBW** ^5^	2,791	338 (12.1%)	1,424	167(11.7%)
**Congenital abnormality** ^6^	2,815	29 (1.0%)	1,432	17 (1.2%)

Data are *n* (percent) or mean (standard deviation), except for gestational age, which is median (interquartile range).

^1^Hb < 125 g/l.

^2^Available data from Benin, Gabon, and Mozambique.

^3^Calculated by Ballard score.

^4^Gestational age < 37 wk.

^5^Birth weight < 2,500 g.

^6^Reported as SAE at delivery or during the follow-up.

### Nutritional Outcomes

There were no significant differences between the groups at any of the scheduled study visits in the proportion of children who were stunted, underweight, wasted, or with severely acute malnutrition. There was a non-significant increase from the first study visit at 1 mo of age to the last study visit at 12 mo of age in the prevalence of children who were stunted or wasted. The proportion of children who were underweight at 12 mo (25.7% in the MQ group and 26.1% in the SP group) was four times greater than the proportion who were underweight at 1 mo of age (7.7% in the MQ group and 6.8% in the SP group, odds ratio 4.31, 95% CI 2.28–3.51, *p* < 0.001) (Table 2).

**Table 2:** Nutritional outcomes of study infants by age and by their mother’s intervention group.

Outcome	MQ Group		SP Group		RR (95% CI)	*p*-Value
	*N*	*n* (Percent)	*N*	*n* (Percent)		
**1 mo**						
Stunting	2,296	268 (11.7%)	1,146	149 (13.0%)	0.90 (0.71–1.09)	0.243
Underweight	2,323	178 (7.7%)	1,150	78 (6.8%)	1.13 (0.86–1.49)	0.369
Wasting	2,250	246 (10.9%)	1,135	120 (10.6%)	1.03 (0.84–1.27)	0.746
Severe acute malnutrition	2,250	67 (3.0%)	1,135	44 (3.9%)	0.77 (0.53–1.12)	0.166
**9 mo**						
Stunting	2,073	268 (12.9%)	1,038	114 (11.0%)	1.19 (0.97–1.47)	0.101
Underweight	2,072	367 (17.7%)	1,040	203 (19.5%)	0.90 (0.77–1.05)	0.198
Wasting	2,069	198 (9.6%)	1,032	105 (10.2%)	0.94 (0.75–1.17)	0.563
Severe acute malnutrition	2,069	76 (3.7%)	1,032	35 (3.4%)	1.08 (0.73–1.60)	0.703
MUAC < 115 cm	2,120	34 (1.6%)	1,062	18 (1.7%)	0.95 (0.52–1.73)	0.867
**12 mo**						
Stunting	2,028	310 (15.3%)	1,045	164 (15.7%)	0.98 (0.82–1.16)	0.798
Underweight	2,028	521 (25.7%)	1,041	272 (26.1%)	0.98 (0.86–1.11)	0.720
Wasting	2,028	242 (11.9%)	1,032	136 (13.2%)	0.89 (0.74–1.09)	0.257
Severe acute malnutrition	2,028	77 (3.8%)	1,032	41 (4.0%)	0.94 (0.65–1.36)	0.748
MUAC < 115 cm	2,091	22 (1.1%)	1,071	14 (1.3%)	0.79 (0.39–1.59)	0.512

mITT analysis adjusted by country. Outcome definitions: stunting, height-for-age *z*-score < −2 SD; underweight, weight-for-age *z*-score < −2 SD; wasting, weight-for-height *z*-score < −2 SD; severe acute malnutrition, weight-for-age *z*-score < −3 SD. MUAC, mid-upper arm circumference.

### Psychomotor Development

Among infants born to women in the MQ group, there was an increased risk of being unable to stand without help, walk without support, and bring solid food to the mouth at 9 mo of age compared to those children born to women in the SP group (RR 1.07, 95% CI 1.00–1.14, *p* = 0.040; RR 1.10, 95% CI 1.01–1.21, *p* = 0.039; RR 1.32, 95% CI 1.03–1.70, *p* = 0.031, respectively), but not at 1 and 12 mo. No other significant differences were observed in the psychomotor development milestones assessed at the study visits (Table 3). Similar differences in the same psychomotor development items at 9 mo were found in both the according-to-protocol (ATP) and mITT analysis. In addition, no other associations were found in the rest of the items assessed or at the other study visits for the ATP group (S6 Table).

**Table 3:** Psychomotor development of children by age and by their mother’s study group.

Development Outcome	MQ Group		SP Group		RR (95% CI)	*p*-Value
	*N*	*n* (Percent)	*N*	*n* (Percent)		
**1 mo**						
Unable to move four extremities symmetrically	2,327	2 (0.1%)	1,153	0 (0.0%)	—	0.319^1^
Abnormal muscle tone	2,324	7 (0.3%)	1,153	0 (0.0%)	—	0.104^1^
Unable to follow objects	2,328	521 (22.4%)	1,154	266 (23.1%)	0.97 (0.86–1.09)	0.569
No response to sounds	2,328	196 (8.4%)	1,153	85 (7.4%)	1.12 (0.90–1.04)	0.320
No response to smiles	2,328	793 (34.1%)	1,154	365 (31.6%)	1.07 (0.98–1.18)	0.122
**9 mo**						
Unable to sit without leaning	2,086	17 (0.8%)	1,045	6 (0.6%)	1.43 (0.56–3.66)	0.460
Unable to crawl	2,087	190 (9.1%)	1,045	90 (8.6%)	1.06 (0.84–1.35)	0.619
Unable to stand without help	2,084	1,231 (59.1%)	1,045	576 (55.1%)	1.07 (1.00–1.14)	0.040
Unable to walk without support	2,085	881 (42.3%)	1,045	403 (38.6%)	1.10 (1.01–1.21).	0.039
Unable to grasp small objects	2,086	30 (1.4%)	1,045	16 (1.5%)	0.94 (0.51–1.71)	0.831
Unable to do palm grasp	2,084	13 (0.6%)	1,045	12 (1.1%)	0.54 (0.25–1.19)	0.126
Unable to move objects from one hand to the other	2,086	86 (4.1%)	1,044	42 (4.0%)	1.04 (0.73–1.49)	0.826
No turn at voice	2,086	9 (0.4%)	1,045	4 (0.4%)	1.13 (0.35–3.65)	0.843
Unable to say any word	2,085	660 (31.7%)	1,045	327 (31.3%)	1.00 (0.91–1.10)	0.950
Unable to bring solid food to his/her mouth	1,966	195 (9.9%)	976	74 (7.6%)	1.32 (1.03–1.70)	0.031
**12 mo**						
Unable to walk	2,053	903 (44.0%)	1,053	449 (42.6%)	1.03 (0.94–1.12	0.547
Unable to do pincer grasping	2,051	77 (3.8%)	1,054	26 (2.5%)	1.52 (0.98–2.35)	0.061
Unable to understand orders	2,052	184 (9.0%)	1,054	96 (9.1%)	0.96 (0.77–1.20)	0.719
Unable to say some words	2,046	265 (13.0%)	1,054	124 (11.8%)	1.11 (0.91–1.35)	0.322
Unable to drink from a cup	2,048	206 (10.1%)	1,052	94 (8.9%)	1.12 (0.88–1.41)	0.353

mITT analysis adjusted by country.

^1^Fisher’s exact test.

### Morbidity and Mortality

Throughout the study follow-up, the most common SAEs reported were infectious illnesses, and among these, the most frequently reported were malaria, pneumonia, and neonatal sepsis, with no significant differences between the two groups (S2 and S3 Tables). Nor was there any significant difference between the study groups in the incidence of clinical malaria (0.14 and 0.15 episodes per person per year at risk in the MQ and SP groups, respectively, *p* = 0.594). The incidence of outpatient visits was 0.65 episodes per person per year at risk in both groups. Hospital admissions were also very similar in children born to women who received MQ versus SP, with incidences of 0.09 and 0.10 admissions per person per year at risk, respectively. The infant mortality rate was 26.9 deaths per 1,000 live births per year at risk in both groups (Table 4). Overall, 61% of infant deaths occurred in the neonatal period, of which 43% took place in the first week of life, with no significant difference between the MQ and SP groups. The most common causes of death, with similar proportions in both groups, were non-cause-specific disorders (29%), infectious diseases (25%), respiratory diseases (21%), perinatal complications (9%), blood disorders (6%), congenital abnormalities (5%), and neurological diseases (3%) (S4 Table).

**Table 4:** Incidence of clinical malaria, hospital admissions, outpatient visits, and mortality in study infants by their mother’s intervention group.

Outcome	MQ Group		SP Group		Relative Rate (95% CI)	*p*-Value
	*N*/PYAR^4^	Incidence	*N*/PYAR	Incidence		
Malaria	377/2,712.9	0.14	205/1,388.7	0.15	0.95 (0.81–1.13)	0.594
Anemia^1^	1,898/3,923.4	0.48	919/1,976.8	0.46	1.04 (0.96–1.12)	0.354
Hospital admissions	206/2,401.3	0.09	123/1,221.2	0.10	0.85 (0.68–1.06)	0.251
Outpatient visits	4,261/6,514.8	0.65	2,170/3,327.9	0.65	1.00 (0.95–1.05)	0.886
Mortality	65/2,413.4	0.03	33/1,223.6	0.03	1.00 (0.66–1.51)	0.985

mITT analysis adjusted by country.

^1^Hb < 110 g/l; data available only among children who were tested for malaria.

PYAR, person-years at risk.

## Discussion

This study assessed the effects of MQ as IPTp compared to that of SP on health outcomes in cohorts of infants from four African countries (Benin, Gabon, Mozambique, and Tanzania). Nearly 4,000 infants born to women participating in a large randomized controlled trial assessing the safety and efficacy of IPTp with MQ compared to SP were followed until 12 mo of age. At all study visits, the prevalence of undernutrition in infants was similar between the study groups. Being able to stand without help, walk without support, and bring solid food to the mouth when assessed at 9 mo of age were the only developmental items found to be less frequently done by infants in the MQ group compared to those in the SP group. No significant differences were observed in any of the other developmental items assessed in the study visits at 1 mo, 9 mo, and 12 mo of age. Whether this result could be a chance finding due to multiple testing or related to maternal exposure to MQ during pregnancy would need to be further investigated. No significant differences were observed in the incidences of clinical malaria, anemia, hospital admissions, outpatient visits, or mortality between the groups.

There is very limited data on the impact of MQ in pregnancy on the infant’s health status. Previous trials evaluating the safety and efficacy of MQ in pregnancy in the African region assessed only pregnancy outcomes or followed up children only until 1 mo after birth [37–39]. Only two trials—one carried out in the Thai–Burmese border area comparing MQ with placebo for malaria prophylaxis in pregnant Karen women and the other, in the same area, comparing MQ combined with AS with quinine for malaria treatment—have reported children’s outcomes for the first 24 and 12 mo of life, respectively [27,28]. However, in the first study, MQ was given at a prophylactic dosage (2.5 mg of base/kg/wk), making it difficult to extrapolate the findings to higher treatment dosages. In the other study, the small sample size was insufficient to appropriately assess differences between groups. In the first trial, weight and weight gain at months 1, 3, 6, and 12 were similar in children born to women who received MQ and those born to women who received placebo [27]. In the second trial, infant growth outcomes were not assessed. An analysis of a case series of 72 American female soldiers who took weekly MQ prophylaxis without prior knowledge of their pregnancy status found that two out of the 13 live-born infants with available data were small for their ages [40]. Other than these studies, most of the safety data in children born to women who took MQ while pregnant come from the post-marketing surveillance system of the manufacturer (dominated by exposure as chemoprophylaxis) and from studies in Southeast Asia, none of which assessed the impact of MQ during pregnancy in children older than 1 mo of age [40–44].

Though controversy exists as to which indicator best defines undernutrition, the use of stunting (low height for age) seems to be the most appropriate, since it is a largely irreversible outcome and has long-term effects in individuals and societies [45]. The prevalence of stunting observed at 12 mo of age in this study is comparable to that found in other studies carried out in African populations [47–49]. More than a quarter of the children in both groups were underweight at 12 mo of age, which is consistent with figures reported from other low-income settings [50–53]. On the other hand, in both groups the prevalence of underweight increased with age from 1 to 12 mo. Although exclusive breast-feeding is considered sufficient to provide adequate nutrition and immunological protection to infants during the first 6 mo, the likely inadequacy, both in quantity and nutritional quality, of weaning foods, together with exposure to infectious diseases, may compromise the infants’ growth onwards [54–57].

While in high-income countries many tools have been developed and validated to evaluate children’s psychomotor development, there is a shortage of appropriate assessment tools for populations living in low-income settings [58,59]. In this study, an adapted and simplified test was used, which might be difficult to compare with that used in other studies [30]. In the two previously described trials carried out in Thailand, no significant differences were found in neurological development assessed by the Denver Developmental Screening Test at 24 mo of age between the children of women who received MQ-AS versus quinine for the treatment of malaria in pregnancy, and mean ages to sit and to crawl were significantly younger in the children of women given MQ versus placebo as malaria prophylaxis [27,28,60]. In contrast, in the current study, there was a higher proportion of children unable to perform certain developmental items at 9 mo of age in the MQ group compared to the SP group, though it is likely that this may be due to multiple testing rather than to true differences between the groups.

Several studies have evidenced an association of maternal malaria, especially placental infection, with increased risk of clinical malaria and overall mortality in infants [1,2,4,6,11,61–63]. Although pregnant women who received IPTp with MQ had a lower incidence of clinical malaria and placental infection than those who received SP, the incidence of malaria and the all-cause mortality rate among their children were similar between the two groups and consistent with results reported from other studies in sub-Saharan Africa and are similar to those estimated in the region for the same age group [2,14,29,38,39,64]. In the only trial assessing the impact of MQ in pregnancy on malaria and all-cause mortality in children, the mortality rate and incidence of malaria episodes were comparable in both groups (malaria prophylaxis with either MQ or placebo) [27].

The main strength of this study is that it provides carefully collected and detailed information on nutritional and developmental outcomes in a large number of African infants born to women who randomly received either MQ or SP as IPTp for malaria prevention. This is of relevance given the limited data available on the effect of MQ in pregnancy on the infant’s health. No previous trials to our knowledge have assessed the effect of MQ administration in African pregnant women on the infant’s health. The results are also important because drug combinations containing MQ are currently recommended for malaria treatment in pregnancy, and MQ alone is recommended for prophylaxis in pregnant women traveling to endemic countries. A limitation of the study was that more than a quarter of infants did not complete the study, though only 9% of them were lost to follow-up. It has been previously noted that loss to follow-up in infants is a potential operational challenge for implementation of preventive programs and for performance of observational studies in this age group in sub-Saharan Africa [65–67]. The proportion of deaths in the first year of life in our study was similar in the two study groups and lower than the 6% UNICEF estimate for the African region [68]. It is common practice in rural Africa for women to move to their parents’ house to give birth, often in a different village. After delivery they move back to their home village, which might explain the migrations during infancy that caused some participants to not complete the study. The baseline characteristics of infants who did not complete the study were similar to those of children who did so, except for the country distribution, which could be explained by the particularities of each site’s follow-up methodology. We did not find any significant difference regarding newborn physical characteristics between those infants who completed the study and those who did not, supporting that infants who did not complete the study did not have an underlying condition or an increased frequency of adverse events that could have influenced the results in one way or another. On the other hand, the open-label design might have led to biases in the assessment of the outcomes, especially of psychomotor development, since MQ is often associated with neurological adverse events. However, it is difficult to rule out or confirm that this could have affected the study results. Children were uniquely identified by a different study number from that of the mother, and were examined by different study personnel, who did not have access to the mother’s study arm allocation. Only study personnel who administered the study interventions to the women were aware of the drug they were providing to the participants, and in general women did not know whether they had received MQ or SP.

In conclusion, these results indicate that administration of 15 mg/kg of MQ as IPTp compared to SP as IPTp in pregnant women is not associated with increased risks of infant mortality, morbidity, and undernutrition. This is of particular relevance considering that antimalarial drug combinations containing MQ are currently recommended for malaria treatment in pregnancy, and MQ alone is recommended for prophylaxis in pregnant women traveling to endemic countries [22,23]. In the current study there was a higher proportion of children unable to perform certain developmental items at 9 mo of age in the MQ group compared to the SP group. Though this finding is interesting and may call for further studies in children whose mothers are exposed to MQ during pregnancy, it cannot be ruled out that it might be explained by the multiple testing or open design of the study.

## Supporting Information

S1 Table Local regulatory authorities and national ethics review committees. (PDF)

S2 Table SAEs in children by system organ class and by their mother’s study group. (PDF)

S3 Table Medical Dictionary for Regulatory Activities term codification of infection and infestation SAEs in children by their mother’s study group. (PDF)

S4 Table Infant causes of death by system organ class and by their mother’s study group. (PDF)

S5 Table Comparison of baseline characteristics at delivery of children who completed the study and children who did not complete the study (excluding deaths). (PDF)

S6 Table Psychomotor development assessment in the ATP group. (PDF)

S7 Table Study outcomes by MQ dose regimen. (PDF)

S1 Text Study protocol. (PDF)

S2 Text CONSORT statement. (PDF)

S3 Text STROBE statement. (PDF)

S4 Text Trial analytical plan. (PDF)

S5 Text Specific analytical plan for follow-up of infants. (PDF)

S6 Text Ethics approval. (PDF)

## Abbreviations

AS: artesunate
ATP: according-to-protocol
Hb: hemoglobin
IPTp: intermittent preventive treatment of malaria in pregnancy
LBW: low birth weight
mITT: modified intention-to-treat
MQ: mefloquine
RR: relative risk
SAE: serious adverse event
SP: sulfadoxine-pyrimethamine
