# Supplementary material for: Mortality, Morbidity, and Developmental Outcomes in Infants Born to Women Who Received Either Mefloquine or Sulfadoxine-Pyrimethamine as Intermittent Preventive Treatment of Malaria in Pregnancy: A Cohort Study
Source: PLoS Med. 2016 Feb 23;13(2):e1001964. doi: 10.1371/journal.pmed.1001964 (PMC4764647; doi:10.1371/journal.pmed.1001964)
Supplement: S1 Table — (PDF) [file pmed.1001964.s001.pdf]

**Table S1. Local regulatory authorities and national ethical review boards**

| Country | Review Board |
|---|---|
| **Benin** | *Comité d' Ethique de l'Université Abomey Calavi* |
| **Gabon** | *Comité d'Ethique Régional Indépendant de Lambaréne* |
| **Mozambique** | *Ministério de Saúde*<br>*Comité Nacional de Bioética para a Saúde* |
| **Tanzania** | *Institutional Review Board*<br>*National Institute for Medical Research Review Board*<br>*Tanzania Food and Drug Association* |
