# Supplementary material for: Mortality, Morbidity, and Developmental Outcomes in Infants Born to Women Who Received Either Mefloquine or Sulfadoxine-Pyrimethamine as Intermittent Preventive Treatment of Malaria in Pregnancy: A Cohort Study
Source: PLoS Med. 2016 Feb 23;13(2):e1001964. doi: 10.1371/journal.pmed.1001964 (PMC4764647; doi:10.1371/journal.pmed.1001964)
Supplement: S2 Table — (PDF) [file pmed.1001964.s002.pdf]

Table S2. Serious adverse events of children by system organ class and by their mother's study group

Serious adverse event by system organ class (SOC)	MQ[1] (N=2815)		SP[2] (N=1432)	
	%	n	%	n
Infectious diseases	7.2	202	7.6	109
Non cause-specific disorders	1.5	41	1.5	21
Respiratory diseases	1.1	31	1.0	14
Congenital abnormalities	0.8	23	1.0	14
Blood disorders	0.4	11	0.2	3
Perinatal complications	0.5	13	0.9	13
Metabolism and nutrition disorders	0.2	5	0.0	0
Neurological diseases	0.1	4	0.3	4
Endocrine disorders	0.0	1	0.0	0
Eye disorders	0.0	1	0.0	0
Gastrointestinal disorders	0.2	7	0.2	3
Investigations	0.1	3	0.1	2
Injury, poisoning and procedural complications	0.0	1	0.0	0
Renal and urinary diseases	0.0	0	0.1	1

[1]mefloquine [2] suphadoxine-pyrimethamine
