# Supplementary material for: Mortality, Morbidity, and Developmental Outcomes in Infants Born to Women Who Received Either Mefloquine or Sulfadoxine-Pyrimethamine as Intermittent Preventive Treatment of Malaria in Pregnancy: A Cohort Study
Source: PLoS Med. 2016 Feb 23;13(2):e1001964. doi: 10.1371/journal.pmed.1001964 (PMC4764647; doi:10.1371/journal.pmed.1001964)
Supplement: S3 Table — (PDF) [file pmed.1001964.s003.pdf]

Table S3. Meddra term codification of infections and infestations serious adverse events in the children by their mother's study group

Meddra System Organ Class	Meddra Term	MQ[1] (N=2815)		SP[2] (N=1432)	
		%	n	%	n
Infections and infestations	Malaria	2.4	67	2.5	36
	Sepsis neonatal	1.1	31	0.8	12
	Pneumonia	1.3	36	1.5	21
	Enterocolitis infectious	1.1	32	1.3	18
	Neonatal infection	0.3	7	0.4	6
	Meningitis	0.1	4	0.1	2
	Bronchopneumonia	0.0	1	0.0	0
	Urinary tract infection	0.1	2	0.0	0
	Infection	0.1	2	0.1	1
	Respiratory tract infection	0.2	6	0.2	3
	Bronchiolitis	0.2	6	0.3	4
	Gastroenteritis	0.1	2	0.0	0
	Sepsis	0.1	2	0.1	1
	Cellulitis	0.0	1	0.1	0
	Breast abscess	0.1	2	0.0	0
	Bullous impetigo	0.1	2	0.1	2
	Meningitis pneumococcal	0.0	1	0.0	0
	Abscess soft tissue	0.1	2	0.0	0
	Subcutaneous abscess	0.0	1	0.0	0
	Skin infection	0.1	3	0.0	0
	Omphalitis	0.0	1	0.1	2
	Abscess neck	0.0	0	0.1	1
	Toxoplasmosis	0.0	0	0.1	1
	Upper resp. tract infection	0.0	1	0.0	0
	Pneumococcal sepsis	0.0	0	0.1	1
	Arthritis infective	0.0	0	0.1	1
	Oral candidiasis	0.0	1	0.0	0

[1] mefloquine [2] sulphadoxine-pyrimethamine
