# Supplementary material for: Mortality, Morbidity, and Developmental Outcomes in Infants Born to Women Who Received Either Mefloquine or Sulfadoxine-Pyrimethamine as Intermittent Preventive Treatment of Malaria in Pregnancy: A Cohort Study
Source: PLoS Med. 2016 Feb 23;13(2):e1001964. doi: 10.1371/journal.pmed.1001964 (PMC4764647; doi:10.1371/journal.pmed.1001964)
Supplement: S4 Table — (PDF) [file pmed.1001964.s004.pdf]

Table S4. Infant causes of death by System Organ Class and by their mother´s study group.

System Organ Class	IPTp[1]				Total	
	MQ[2] (N=103)		SP[3] (N=54)		n	%
	n	%	n	%		
Non cause-specific disorders	30	29.1	15	27.8	45	28.7
Infectious diseases	28	27.2	12	22.2	40	25.5
Respiratory diseases	23	22.3	10	18.5	33	21.0
Perinatal complications	7	6.8	7	13.0	14	8.9
Blood disorders	8	7.8	1	1.8	9	5.7
Congenital abnormalities	3	2.9	5	9.3	8	5.1
Neurological diseases	2	1.9	3	5.6	5	3.2
Renal and urinary diseases	0	0.0	1	1.9	1	0.6
Endocrine disorders	1	1.0	0	0.0	1	0.6
Gastrointestinal disorders	1	1.0	0	0.0	1	0.6
Total	103	65.6	54	34.4	157	100.0

[1] Intermittent preventive treatment of malaria in pregnancy [2]mefloquine [3]sulphadoxine-pyrimethamine
