# Supplementary material for: Mortality, Morbidity, and Developmental Outcomes in Infants Born to Women Who Received Either Mefloquine or Sulfadoxine-Pyrimethamine as Intermittent Preventive Treatment of Malaria in Pregnancy: A Cohort Study
Source: PLoS Med. 2016 Feb 23;13(2):e1001964. doi: 10.1371/journal.pmed.1001964 (PMC4764647; doi:10.1371/journal.pmed.1001964)
Supplement: S5 Table — (PDF) [file pmed.1001964.s005.pdf]

Table S5. Comparison of baseline characteristics at delivery of children who completed the study and children who did not complete the study (excluding deaths).

Baseline characteristics		Children who completed study (N=3109)	Children who did not complete the study , excluding deaths (N=972)	P-value
		n (%)	n (%)	
Live births by country (n (%))	Benin	875 (28.1)	162 (16.7)	<0.001
	Gabon	632 (20.3)	334 (34.4)	
	Mozambique	890 (28.6)	189 (19.4)	
	Tanzania	712 (22.9)	287 (29.5)	
Mothers IPTp group MQ[1,2]		2054 (66.1)	654 (67.3)	0.483
Males		1551 (49.9)	481(49.5)	0.794
Weight (g, mean (sd))		3040.51(466.9)	3018.79 (469.85)	0.208
Length (cm, mean (sd))		48.35 (8.8)	48.57 (11.4)	0.568
Head circumference (cm, mean (sd))		33.83 (2.1)	33.67 (2.2)	0.038
Cord blood anemia [3]		344 (11.1)	79 (8.1)	0.026
Cord blood parasitemia blood smear		9 (0.3)	1 (0.1)	0.344
Gestational age (weeks, median(IQR))[4,5]		39.2(38.4;40.4)	40.00 (39.2;40.8)	0.000
Prematurity [4,5,6]		119 (3.8)	38 (3.9)	0.602
Low birth weight [7]		323 (10.4)	109 (11.2)	0.439
Congenital abnormalities [8]		42 (1.4)	17 (1.8)	0.304
Reasons for not completing the study				
Deaths			166 (3.9)	
Withdrawals			256 (6.0)	
Migration			338 (8.0)	
Lost to follow-up			378 (8.9)	

Chi-square test [1] Intermittent treatment of malaria in pregnancy [2] mefloquine [3]haemoglobin <12.5 g/dl [4]available data from Benin, Gabon and Mozambique [5]calculated by Ballard score [6]gestational age<37 weeks [7]birth weight<2500g [8] reported as SAE at delivery and during the follow-up
