# Supplementary material for: Mortality, Morbidity, and Developmental Outcomes in Infants Born to Women Who Received Either Mefloquine or Sulfadoxine-Pyrimethamine as Intermittent Preventive Treatment of Malaria in Pregnancy: A Cohort Study
Source: PLoS Med. 2016 Feb 23;13(2):e1001964. doi: 10.1371/journal.pmed.1001964 (PMC4764647; doi:10.1371/journal.pmed.1001964)
Supplement: S6 Table — (PDF) [file pmed.1001964.s006.pdf]

Table S6. Psychomotor development assessment in the ATP group

ATP group[1]	MQ[2]		SP[3]		RR[4]	(95% CI)	P-VALUE
	N	n (%)	N	n(%)			
**Month 1**							
Not move 4 extremities symmetrically	1805	0	1048	0	-	-	-
Abnormal muscle tone	1804	3 (0.17)	1047	0	-	-	-
Unable to follow objects	1805	395 (21.88)	1048	238 (22.71)	0.97	0.86;1.10	0.605
No response to sounds	1805	145 (8.03)	1047	84 (8.02)	1.09	0.86;1.38	0.474
No response to smiles	1805	598 (33.13)	1048	327 (31.20)	1.10	1.00;1.21	0.070
**Month 9**							
Not able to sit without leaning	1626	12(0.74)	954	6 (0.63)	1.16	0.45;3.03	0.758
Not able to crawl	1627	139 (8.54)	954	77 (8.07)	1.04	0.80;1.35	0.778
Unable to stand without help	1624	973 (59.91)	954	520 (54.51)	1.09	1.01;1.17	0.019
Unable to walk without support	1625	670 (41.23)	954	361 (37.84)	1.12	1.02;1.24	0.024
Unable to grasp small objects	1625	18 (1.11)	954	15 (1.57)	0.80	0.41;1.57	0.515
Unable to do palm grasp	1625	10 (0.62)	954	9 (0.94)	0.72	0.30;1.73	0.458
Unable to move objects from one hand to the other	1625	64 (3.94)	953	41 (4.30)	0.99	0.67;1.44	0.939
Not turn at voice	1625	7 (0.43)	954	4 (0.42)	0.96	0.28;3.31	0.954
Unable to say any word	1625	462 (28.43)	954	296 (31.03)	0.96	0.86;1.07	0.454
Unable to bring solid food to his/her mouth	1524	145 (9.51)	890	75 (8.43)	1.26	0.96;1.65	0.089
**Month 12**							
Unable to walk	1580	713 (45.13)	952	400 (42.02)	1.04	0.95;1.14	0.358
Unable to do pincer grasping	1578	48 (3.04)	953	22 (2.31)	1.33	0.81;2.18	0.266
Unable to understand orders	1579	144 (9.12)	953	78 (8.18)	0.96	0.75;1.22	0.726
Unable to say some words	1575	180 (11.43)	953	110 (11.54)	1.05	0.84;1.31	0.670
Unable to drink from a cup	1577	147 (9.32)	951	88 (9.25)	0.95	0.74;1.22	0.680

[1] According to protocol [2] Mefloquine [3] Sulphadoxine-pyrimethamine [4] Relative risk
