# Supplementary material for: Mortality, Morbidity, and Developmental Outcomes in Infants Born to Women Who Received Either Mefloquine or Sulfadoxine-Pyrimethamine as Intermittent Preventive Treatment of Malaria in Pregnancy: A Cohort Study
Source: PLoS Med. 2016 Feb 23;13(2):e1001964. doi: 10.1371/journal.pmed.1001964 (PMC4764647; doi:10.1371/journal.pmed.1001964)
Supplement: S7 Table — (PDF) [file pmed.1001964.s007.pdf]

Table S6.1. Incidence of malaria, anemia, hospital admissions, outpatient visits and mortality in the first year of life by MQ regimen

Incidences	MQ[1] full-dose		MQ Split-dose		RR[3]	95% CI	P-value
	N/PYAR[2]	Incidence	N/PYAR	Incidence			
Malaria	191/1347.30	0.14	186/1365.64	0.14	1.06	0.86;1.29	0.597
Anemia[4]	858/ 1728.94	0.50	618/1295.12	0.48	1.06	0.97;1.16	0.190
Hospital Admissions	105/1194.55	0.09	101/1206.66	0.08	1.05	0.80;1.39	0.703
Outpatient visits	2265/3378.91	0.67	1996/3135.87	0.64	1.05	0.99;1.11	0.127
Mortality	32/1200.26	0.03	33/1213.17	0.03	0.98	0.60;1.59	0.921

Intention to treat analysis adjusted by country [1] Mefloquine [2]Person year at risk [3]Relative Rate [4] Hb<11g/dl, data available only among children who were tested for malaria.

Table S6.2. Nutritional status in infants at month 1, 9 and 12 by MQ regimen

ITT GROUP[1]	MQ full-dose[2]		MQ split-dose		RR[3]	95% CI	P-value
	N	n (%)	N	n (%)			
**Month 1**							
Stunting (HAZ<-2SD)[4]	1148	134 (11.67)	1148	134 (11.67)	1.01	0.80;1.27	0.959
Underweight (WAZ<-2SD)[5]	1159	87 (7.51)	1164	91 (7.82)	0.96	0.71;1.29	0.775
Wasting (WHZ<-2SD)[6]	1130	120(10.62)	1120	126 (11.25)	0.94	0.74;1.19	0.618
Severe Acute Malnutrition[7]	1130	31 (2.74)	1120	36 (3.21)	0.85	0.53;1.37	0.511
**Month 9**							
Stunting	1031	134 (13.00)	1042	134 (12.86)	1.02	0.81;1.28	0.879
Underweight	1033	185 (17.91)	1039	182 (17.52)	1.02	0.84;1.22	0.868
Wasting	1030	109 (10.58)	1039	89 (8.57)	1.22	0.94;1.59	0.137
Severe Acute Malnutrition	1030	39 (3.79)	1039	37 (3.56)	1.05	0.67;1.65	0.827
MUAC <115cm[8]	1055	13 (1.23)	1065	21 (1.97)	0.62	0.30;1.28	0.198
**Month 12**							
Stunting	1021	150 (14.69)	1007	160 (15.89)	0.95	0.77;1.16	0.598
Underweight	1026	271 (26.41)	1002	250 (24.95)	1.05	0.91;1.22	0.486
Wasting	1021	126 (12.34)	1007	116 (11.52)	1.06	0.84;1.34	0.635
Severe Acute Malnutrition	1021	47 (4.60)	1007	30 (2.98)	1.54	0.97;2.44	0.068
MUAC <115	1055	8 (0.76)	1036	14 (1.35)	0.57	0.24;1.37	0.208

[1]Intention to treat analysis adjusted by country [2]Mefloquine [3]Relative risk [4]Height for age z-score [5] Weight for age z-score [6] Weight for height z-score [7] WAZ<-3SD [8]Middle upper arm circumference

Table S6.3. Psychomotor development assessment in infants at month 1, 9 and 12 by MQ regimen

ITT group	MQ full-dose[2]		MQ split-dose		RR[3]	95% CI	p-value
	N	n (%)	N	n(%)			
**Month 1**							
Not move 4 extremities symmetrically	1162	0	1165	2 (0.17)	-	-	-
Abnormal muscle tone	1162	3 (0.26)	1162	4 (0.34)	0.74	0.17;3.27	0.686
Unable to follow objects	1162	271 (23.32)	1166	250 (21.44)	1.10	0.96;1.26	0.177
No response to sounds	1162	96 (8.26)	1166	100 (8.58)	0.99	0.77;1.26	0.926
No response to smiles	1162	412 (35.46)	1166	381 (32.68)	1.09	0.98;1.21	0.101
**Month 9**							
Not able to sit without leaning	1038	10 (0.96)	1048	7 (0.67)	1.44	0.53;3.89	0.475
Not able to crawl	1039	90 (8.66)	1048	100 (9.54)	0.91	0.69;1.20	0.501
Unable to stand without help	1037	600 (57.86)	1047	631 (60.27)	0.96	0.89;1.03	0.267
Unable to walk without support	1037	431 (41.56)	1048	450 (42.94)	0.97	0.88;1.07	0.568
Unable to grasp small objects	1037	16 (1.54)	1049	14 (1.33)	1.15	0.57;2.34	0.693
Unable to do palm grasp	1036	7 (0.68)	1048	6 (0.57)	1.18	0.40;3.52	0.765
Unable to move objects from one hand to	1037	43 (4.15)	1049	43 (4.10)	1.02	0.68;1.55	0.917
Not turn at voice	1037	3 (0.29)	1049	6 (0.57)	0.50	0.13;2.01	0.331
Unable to say any word	1037	331 (31.92)	1048	329 (31.39)	1.00	0.90;1.12	0.944
Unable to bring solid food to his/her	979	91 (9.30)	987	104 (10.54)	0.89	0.69;1.16	0.397
**Month 12**							
Unable to walk	1036	476 (45.95)	1017	426 (41.89)	1.10	0.99;1.21	0.067
Unable to do pincer grasping	1035	33 (3.19)	1016	44 (4.33)	0.76	0.49;1.20	0.239
Unable to understand orders	1035	80 (7.73)	1017	104 (10.23)	0.77	0.59;0.99	0.044
Unable to say some words	1033	137 (13.26)	1013	128 (12.64)	1.05	0.84;1.32	0.646
Unable to drink from a cup	1033	113 (10.94)	1015	93 (9.16)	1.20	0.92;1.56	0.172

[1]Intention to treat analysis adjusted by country [2] Mefloquine [3]Relative risk [4] Fisher exact test
