# Supplementary material for: Mortality, Morbidity, and Developmental Outcomes in Infants Born to Women Who Received Either Mefloquine or Sulfadoxine-Pyrimethamine as Intermittent Preventive Treatment of Malaria in Pregnancy: A Cohort Study
Source: PLoS Med. 2016 Feb 23;13(2):e1001964. doi: 10.1371/journal.pmed.1001964 (PMC4764647; doi:10.1371/journal.pmed.1001964)
Supplement: S1 Text — (PDF) [file pmed.1001964.s008.pdf]

Analysis Plan

For the MiPPAD trial 1

Author:	MiPPAD Stats-WG
Version:	Final Version 1.0
Date:	10/Jun/2013
Title:	Evaluation of the safety and efficacy of Mefloquine as Intermittent Preventive Treatment for malaria in Pregnancy
Acronym:	MiPPAD (MOT1)

Contents

1 Introduction 6

2 Trial 1 objectives and endpoints 6

2.1 Primary 6

2.2 Secondary 6

2.3 Exploratory 7

3 Study design overview 7

4 Population of analysis 10

4.1 According to protocol (ATP) 10

4.2 Intention to Treat (ITT) 10

4.3 Safety 10

5 Statistical methods 11

6 Skeleton of tables and graphs 14

6.1 Trial profile (ATP, ITT, Safety) 14

6.2 Baseline characteristics (ATP, ITT, Safety) 16

6.3 Efficacy (ATP Adjusted, ATP crude, ITT) 16

6.3.1 Primary Endpoint 17

6.3.2 Secondary endpoints 19

6.4 Safety (Safety, ITT) 21

6.5 Tolerability (Safety, ITT) 23

7 Individual data listings 24

List of Abbreviations

MiPPAD Malaria in Pregnancy Preventive Alternative Drugs

MQ_f Mefloquine given as full-dose

MQ_s Mefloquine given as an split dose

AP Analysis Plan

ATP According to protocol

EPI Expanded program for immunization

IPTp Intermittent preventive treatment during pregnancy

IQR Interquartilic range

ITT Intention to treat

LBW Low Birth Weight (<2500g)

LLITNs Long lasting insecticide treated nets

MQ Mefloquine

MUAC Mid-upper arm circumference

RR Risk Ratio

RRate Relative Rate

SP Sulphadoxine-Pyrimethamine

List of Tables

1 Maternal visits and procedures schedule . . . 8
2 Infants visits and procedures schedule . . . 9
3 Trial profile . . . 14
4 Reasons for withdrawn before dose 1 . . . 14
5 Reasons for withdrawn before dose 2 . . . 14
6 Reasons for withdrawn before delivery . . . 15
7 Reasons for withdrawn before completing follow-up . . . 15
8 Baseline characteristics . . . 16
9 Prevalence of low birth weight by treatment group . . . 17
10 Prevalence of low birth weight by country . . . 17
11 Low birth weight prevalence BY treatment arm . . . 17
12 Secondary endpoints (prevalence) . . . 19
13 Secondary endpoints (continous variables) . . . 19
14 Secondary endpoints (incidence) . . . 20
15 Secondary endpoints (endpoints for neonatal mortality) . . . 20
16 Summary of serious adverse events . . . 21
17 Serious adverse events by MedRA term . . . 21
18 Prevalence of adverse pregancy outcomes . . . 21
19 Adverse events . . . 22
20 Adverse events related to medication . . . 22
21 Tolerability . . . 23

List of Figures

1 Rules for Non-inferiority 11
2 Rules for Superiority 12
3 Efficacy by country 18

1 Introduction

This Analysis Plan (AP) provides detailed description of the descriptive (with the skeleton of the tables) and inferential statistical analysis for the efficacy and safety data collected for mothers in the Trial 1 of the MiPPAD study. Separate APs for children and exploratory analysis are available.

Any planned modification of the analysis should be documented in this AP.

2 Trial 1 objectives and endpoints

2.1 Primary

1. Prevalence of low birth weight (LBW) newborns (<2500g).

2.2 Secondary

1. Peripheral maternal parasitaemia at delivery (microscopic and submicroscopic[1])
2. Prevalence of *P. falciparum* parasitaemia in cord blood (microscopic and submicroscopic[1])
3. Prevalence of placental *P. falciparum* infection (histology or blood smear, microscopy and submicroscopic[1])
4. Mean maternal haemoglobin (g/dL) at delivery
5. Prevalence of maternal anaemia at delivery (< 11g Hb/dL)
6. Prevalence of severe maternal anaemia at delivery (< 7g Hb/dL)
7. Prevalence of neonatal anemia (Hb<12.5 g/dL in case of cord blood, or Hb <13 g/dL in case of peripheral blood)
8. Mean birth weight (in grams)
9. Prevalence of prematurity
10. Number of stillbirths
11. Number of miscarriages
12. Frequency of congenital malformations.
13. Mean gestational age at birth (assessed by Ballard score in the newborn)
14. Incidence of vomiting
15. Incidence of dizziness
16. Frequency of drug adverse reactions
17. Incidence of clinical malaria during pregnancy
18. Incidence of overall maternal admissions/outpatient attendances
19. Neonatal and infant mortality rate

[1] Only in a subsample of participants

20. Incidence of clinical malaria in the first year of life

21. Peripheral maternal parasitaemia 1 month[2] after end of pregnancy (microscopic and submicroscopic[1])

2.3 Exploratory

1. Frequency of congenital malaria

2. Prevalence of Malaria, development and physicomotor development of children

3. Safety (SAEs) in children

4. Pharmacovigilance (to be presented in a separate document)

5. . . . *Exploratory endpoints (to be completed)*

3 Study design overview

This is a randomized open-label superiority 3 arms trial to compare 2-dose MQ with two dose of SP as IPTp in the prevention of the adverse effects of malaria during pregnancy and to compare MQ tolerability of 2 different MQ administration regimens. The three arms of the study are:

1. IPTp with SP (SP)

2. IPTp with MQ given as full dose (MQ_f)

3. IPTp with MQ given as an split dose (MQ_s)

The table 1 shows the visits and procedures schedules to the mother and the table 2 shows the visits and procedures schedule to the child.

[2]At visit 4, aproximately one month after end of pregnancy

Table 1: Maternal visits and procedures schedule

Study Procedures	Visit 1 Enrollment	Visit 2	Visit 3 Delivery	Visit 4 1 Month	Unscheduled visits	Household visits
Inclusion/Exclusion criteria	X					
Written infomed consent	X					
Demographics/Medical history	X					
Record of concomitant medication/ Adverse events	X	X	X	X	X	
Physical examination / Clinical	X				X	
Gestational Age	X	X	X		X	
Temperature				X	X	
Blood Pressure			X		X	
Weight	X					
Height	X					
MUAC	X					
IPTp Administration	X	X				
RPR test	X					
Blood smear	*	*	X	X	*	
Haemoglobin test	X		X	X		
Peripheral venous blood (mother)			X			
Cord blod sample			X			
Placental biopsy			X			
Placental impression smears			X			
Drug tolerability assesment	X	X				X
LLITNs	X	†	†	†	†	†

* Only in women passively reporting sick AND presenting with malaria related signs/symptoms (fever $\geq$ 17.5°C).
†Assessment of compliance with LLITNs.

Table 2: Infants visits and procedures schedule

Study Procedure	At Birth	1 month§	9 months*	12 months*	Unscheduled visits
Medical history	X	X	X		X
Physical examination	X	X	X	X	X
Psychomotor development assesment	X	X	X	X	
MUAC		X	X	X	X
Weight	X	X	X	X	
Height	X	X	X	X	
Temperature	X	X	X	X	
Blood smear	X	†	†	†	
Haemoglobin test	X	†	†	†	

* Household visits in case they do not attend the scheduled visits at study health facilities.
†Only if fever ($\geq 37.5°C$) or history of fever in the past 24 hours or signs suggestive of malaria.
§First visit will be scheduled 1 month after birth or coinciding with first EPI visit

4 Population of analysis

4.1 According to protocol (ATP)

This population includes all women who fulfill all the inclusion-exclusion criteria and took the two IPTp doses, received the LLITNs and from whom data is available for the analysis.

It will be excluded from the ATP analysis if :

- No fulfill inclusion criteria
- No data on outcome
- No two complete doses of IPTp
- Only half dose of MQ_s
- More than 48 hours for second dose of MQ_s
- Less than 4 weeks (28 days) between doses of IPT (for MQ_s between first half dose)
- Less tablets than specified in the protocol
- Wrong study drug medication
- Weight recorded more than 7 days after delivery
- Do not receive LLITN
- Delivery of twins
- HIV infection in pregnancy

4.2 Intention to Treat (ITT)

This population includes all randomized women. Following the intention-to-treat principle, patients will be analyzed according to the preventive treatment they were assigned to at randomization. This population is the target for the analysis of efficacy.

4.3 Safety

This population includes all patients who received at least a dose of IPT (or at list first half on the MQ_s) and had at least one post-baseline safety assessment. Patients will be analyzed according to the preventive treatment assigned. This is the target population for the safety and tolerability analysis.

5 Statistical methods

For the primary analysis of efficacy, the statistical analysis will follow a sequential approach. First, hypotheses of non-inferiority between the proportion of LBW in the MQ groups and the SP group will be tested assuming as equivalence a 25% reduction or greater in LBW prevalence in the MQ groups. If non-inferiority between MQ and SP is achieved, then an analysis for superiority will be conducted comparing the MQ and SP groups using a two-sided test. Given the long half life of MQ, it has been assumed that MQ efficacy to prevent LBW will be equal in the full dose and split dose groups.

Interpretation of non-inferiority is based on the confidence intervals (CI). If the upper limit of the confidence interval (CI) of the Relative Risk (RR) MQ/SP is below 1.25, (25% of the RR) the MQ arms will be considered as non-inferior compared with SP (See figure 1). In case the upper limit of the CI is above 1.25, the trial is inconclusive as the non-inferiority hypothesis can't be rejected. If both the lower limit of the CI is below 1, the MQ treatment is considered superior to SP (See figure 2).

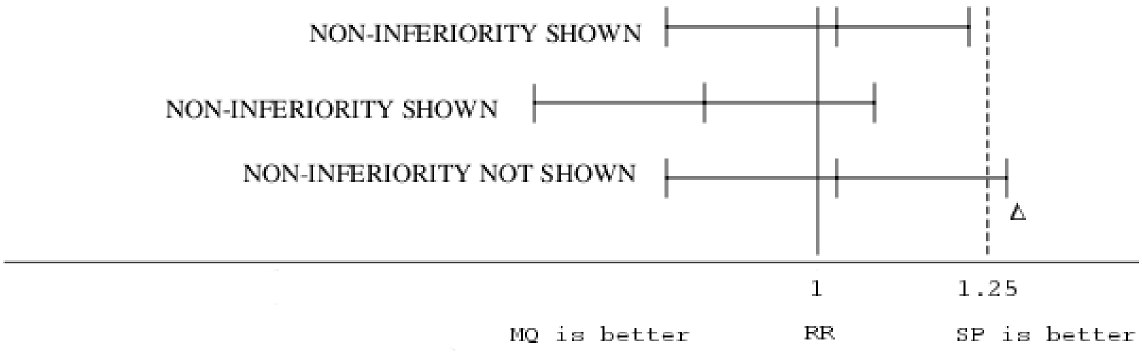

Figure 1: Rules for Non-inferiority

No adjustment of p-values are necessary to this switch as the "delta" has been pre-specified, and then the interpretation based on confidence intervals can support the interpretation of non-inferiority. It is acceptable to calculate the p-value associated with a superiority test and to evaluate whether this is sufficiently small to reject convincingly the hypothesis of no difference. There is no multiplicity that affect this interpretation because in statistical terms it corresponds to a simple closed test procedure (Committee for Proprietary Medicinal Products CPMP, Points to consider on switching between superiority and non-inferiority, CMP/EWP/482/99, July 2000).

Proportions will be compared between groups using fisher exact test and presented as Risk ratio (RR) or reduction of the RR ($1 - RR * 100\%$) if RR lower than 1. Adjustment for covariates and possible confounder are done using binomial regression with log link, and using robust estimates of the covariance (huber method) using the method proposed by Zou (Zou G., A modified poisson regression approach to prospective studies with binary data. American Journal of Epidemiology. 2004;159(7):702-706).

Continuous variables will be compared between groups and adjusted for covariates and possible confounders using ordinary least square regression. Variables will be transformed to the logarithm scale if normality is improved and result presented as Proportional Difference.

Incidence of clinical malaria, overall admissions and outpatient attendances will be estimated as the number of episodes over the time at risk. Time at risk is estimated as the time from the start of follow up (recruitment in mothers, day of birth in infants) until the end of follow-up (visit one month after

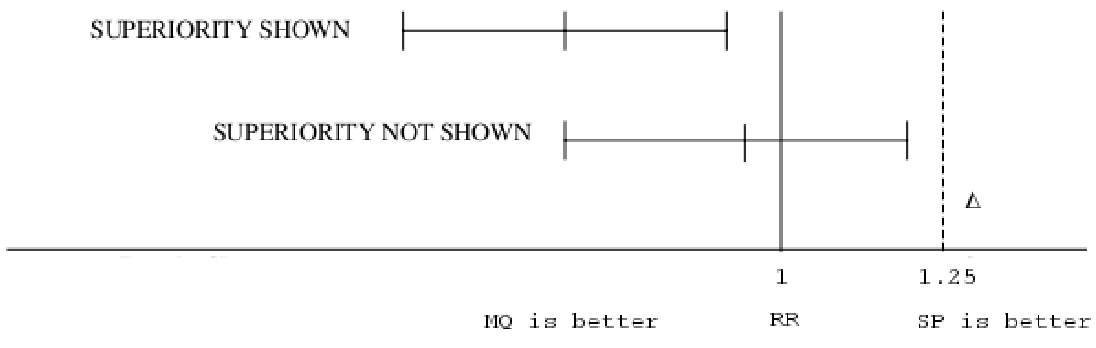

Figure 2: Rules for Superiority

delivery for mothers and visit after 12 months of age in infants) or withdrawal due to censoring or death, whatever occurs first. In order to avoid to count twice the same episode of clinical malaria, subjects will not contribute to the denominator nor the numerator during an arbitrary period of 28 days after an event of clinical malaria is defined. For admissions and outpatient attendances a maximum of one episode per day will be allowed. The total number of events will be compared between groups using negative binomial regression to take into account a possible extra Poisson variation due to different frailty of the subjects. The comparison will be expressed as Relative Rate (RRate)

For the ATP cohorts, in order to allow for center differences, regression models will include site as covariate as well as season. The definition of season will be independent for each site. Interaction between site and treatment to evaluate homogeneity will be estimated using a combined test for the interaction terms. In addition of the adjusted results, the crude results for ATP cohort will be also presented.

For the efficacy, analysis will be presented for ATP and ITT populations. The analysis of ITT population are not adjusted for possible confounders. For Safety and Tolerability, analysis will be presented for Safety and ITT populations, all of them unadjusted. See section 6 for the classification of endpoints efficacy, safety or tolerability.

For the ITT analysis of primary endpoint, which includes all women recruited in the study, missing values for low birth weight will be imputed as follow: For those women with miscarriage, the event will be considered as failure and they will be assigned as having LBW. For those lost to follow-up or withdraw before delivery a sensitivity analysis of worse case scenario assuming all lost of follow-up and withdrawn women having a LBW, best case scenario assuming that none have LBW and worst case scenario assuming as LBW all missing values, will be presented. In case there were countries without malaria events, a sensitivity analysis not including those countries will be also presented.

The assumption that MQ_s and MQ_f have a similar effect on the LBW will be explored by inspection of the 90% confidence intervals of the comparison between MQ_s vs MQ_f in the ATP population. If the assumption of both MQ groups are similar is rejected, then comparison between MQ_s vs SP and MQ_f vs SP will be presented instead.

For the primary outcome, the weight of children born taken after delivery but before the seven days of life are transformed according to the method proposed by *... Greenwod* [3] *(to be completed)* to include in

[3] A regression analysis of the weight and day after delivery

the analysis as if they were taken at birth. Maternal and new born haemoglobines from visit 3(Delivery) not taken in the date of the delivery will not be included in the analysis of these outcomes.

The gestational age will be based on the Ballard score, but in case of missing, it will be based on the gestational age calculated with the fundal height at recruitment.

For the frequency of vomiting, dizziness, prematurity, the proportion of women with those outcomes will be compared using the methods described for proportions before.

For the tolerability, only those vomiting and dizziness judged by the investigators to be related to the medication will be included in the analysis except vomiting during the first 60 minutes after a dose of IPTp that will always be considered related to medication.

6 Skeleton of tables and graphs

6.1 Trial profile (ATP, ITT, Safety)

Table 3: Trial profile

Screened	#		
Group	MQ_s	MQ_f	SP
Randomized	#	#	#
Receives ITN	#	#	#
Withdrawn before dose 1	#	#	#
Dose 1 IPTp	#	#	#
Withdrawn before dose 2	#	#	#
Dose 2 IPTp	#	#	#
Withdrawn before delivery	#	#	#
Delivery	#	#	#
Withdrawn before completing follow-up	#	#	#
Visit after delivery	#	#	#

Table 4: Reasons for withdrawn before dose 1

	MQ_s	MQ_f	SP
Serious Adverse Events	#	#	#
Non Serious Adverse Events	#	#	#
Protocol Violation - No compliance with ATP	#	#	#
Consent withdrawal	#	#	#
Migrated/moved from study area	#	#	#
Lost to follow-up	#	#	#
End of pregnancy / Miscarriage	#	#	#
Others	#	#	#

Table 5: Reasons for withdrawn before dose 2

	MQ_s	MQ_f	SP
Serious Adverse Events	#	#	#
Non Serious Adverse Events	#	#	#
Protocol Violation - No compliance with ATP	#	#	#
Consent withdrawal	#	#	#
Migrated/moved from study area	#	#	#
Lost to follow-up	#	#	#
End of pregnancy / Miscarriage	#	#	#
Others	#	#	#

Table 6: Reasons for withdrawn before delivery

	MQ_s	MQ_f	SP
Serious Adverse Events	#	#	#
Non Serious Adverse Events	#	#	#
Protocol Violation - No compliance with ATP	#	#	#
Consent withdrawal	#	#	#
Migrated/moved from study area	#	#	#
Lost to follow-up	#	#	#
Others	#	#	#

Table 7: Reasons for withdrawn before completing follow-up

	MQ_s	MQ_f	SP
Serious Adverse Events	#	#	#
Non Serious Adverse Events	#	#	#
Protocol Violation - No compliance with ATP	#	#	#
Consent withdrawal	#	#	#
Migrated/moved from study area	#	#	#
Lost to follow-up	#	#	#
Others	#	#	#

6.2 Baseline characteristics (ATP, ITT, Safety)

Table 8: Baseline characteristics

Variable		MQ_s (N=XXX)	MQ_f (N=XXX)	SP (N=XXX)
Country (n(%))	Benin	#(#.#%)	#(#.#%)	#(#.#%)
	Gabon	#(#.#%)	#(#.#%)	#(#.#%)
	Mozambique	#(#.#%)	#(#.#%)	#(#.#%)
	Tanzania	#(#.#%)	#(#.#%)	#(#.#%)
Age (years, mean(sd))		#(#.#)	#(#.#)	#(#.#)
Gravity (n(%))	Primigravidae	#(#.#%)	#(#.#%)	#(#.#%)
	1 to 3 previous pregnancies	#(#.#%)	#(#.#%)	#(#.#%)
	4 or more	#(#.#%)	#(#.#%)	#(#.#%)
Weight (kg, mean(sd))		#(#.#)	#(#.#)	#(#.#)
Height (cm, mean(sd))		#(#.#)	#(#.#)	#(#.#)
MUAC (cm, mean(sd))		#(#.#)	#(#.#)	#(#.#)
Gestational age at recruitment (weeks, median(IQR))		#(#:#)	#(#:#)	#(#:#)
Gestational age in categories	First trimester (0-12 weeks)	#(#.#%)	#(#.#%)	#(#.#%)
	Second trimester (12-24 weeks)	#(#.#%)	#(#.#%)	#(#.#%)
	Third trimester ($\geq$ 25 weeks)	#(#.#%)	#(#.#%)	#(#.#%)
Literate (can read and/or write, n(%))		#(#.#%)	#(#.#%)	#(#.#%)
Syphilis test (Positive, n(%))		#(#.#%)	#(#.#%)	#(#.#%)
Hemoglobin (g/dl, mean(sd))		#(#.#)	#(#.#)	#(#.#)
Anaemia (Hb < 11, n(%))		#(#.#%)	#(#.#%)	#(#.#%)
Severe anaemia (Hb < 7, n(%))		#(#.#%)	#(#.#%)	#(#.#%)

N: Number of subjects in the group, sd: Standard deviation, n: Number of subjects in the category IQR: Interquartilic range, %: Percentage

6.3 Efficacy (ATP Adjusted, ATP crude, ITT)

Efficacy endpoints refers to the effect of MQ on the low birth weight, different forms of malaria infection, malaria disease and anaemia. Analysis are presented adjusted for ATP and also crude for ATP and ITT populations. For the primary endpoint, the sensitivity analysis using best, worst and maximum bias scenarios will be presented. Secondary endpoints will be presented crude for ITT population, and adjusted for country in ATP population. If interaction between country and treatment is significant, secondary endpoints will be also presented by country.

6.3.1 Primary Endpoint

Table 9: Prevalence of low birth weight by treatment group

Variable	Group	N	n(%)	RR (95% CI)	p-value
Low birth weight	MQ<br>SP	###<br>###	## (##.#)<br>## (##.#)	#.## (#.##; #.##)	#.###

RR:Risk Ratio, N: Number of subject per group, n: number of subjects in the category.

Table 10: Prevalence of low birth weight by country

Country	Group	N	n(%)	RR (95% CI)	p-value
Benin	MQ<br>SP	###<br>###	## (##.#)<br>## (##.#)	#.## (#.##; #.##)	#.###
Gabon	MQ<br>SP	###<br>###	## (##.#)<br>## (##.#)	#.## (#.##; #.##)	#.###
Mozambique	MQ<br>SP	###<br>###	## (##.#)<br>## (##.#)	#.## (#.##; #.##)	#.###
Tanzania	MQ<br>SP	###<br>###	## (##.#)<br>## (##.#)	#.## (#.##; #.##)	#.###

RR:Risk Ratio, N: Number of subject per group, n: number of subjects in the category.
Interaction test between Treatment and Country p-value = #.###

Table 11: Low birth weight prevalence BY treatment arm

Variable	Group	N	n(%)	MQ vs SP (90% CI)	p-value	MQ_s vs MQ_f (90% CI)	p-value
Low birth weight	MQ_s<br>MQ_f<br>SP	###<br>###<br>###	## (##.#)<br>## (##.#)<br>## (##.#)	#.## (#.##; #.##)	#.###	#.## (#.##; #.##)	#.###

RR:Risk Ratio, N: Number of subject per group, n: number of subjects in the category.

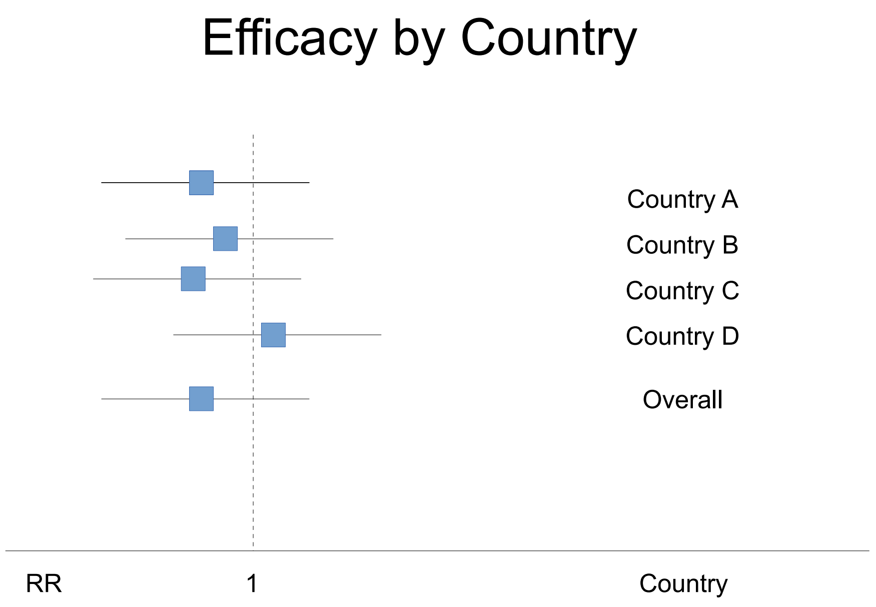

Figure 3: Efficacy by country

6.3.2 Secondary endpoints

Table 12: Secondary endpoints (prevalence)

Variable	Group	N	n(%)	RR (95% CI)	p-value
Peripheral maternal parasitaemia at delivery (Optical microscopy)	MQ<br>SP	###<br>###	## (##.#)<br>## (##.#)	#.## (#.##; #.##)	#.###
Placental *P. falciparum* infection (Histology or blood smear)	MQ<br>SP	###<br>###	## (##.#)<br>## (##.#)	#.## (#.##; #.##)	#.###
Peripheral maternal parasitaemia one month after delivery (Optical Microscopy)	MQ<br>SP	###<br>###	## (##.#)<br>## (##.#)	#.## (#.##; #.##)	#.###
Cord blood parasitaemia at delivery	MQ<br>SP	###<br>###	## (##.#)<br>## (##.#)	#.## (#.##; #.##)	#.###
Maternal anaemia at delivery (Hb < 11 g/dl)	MQ<br>SP	###<br>###	## (##.#)<br>## (##.#)	#.## (#.##; #.##)	#.###
Severe maternal anemia at delivery (Hb < 7 g/dl)	MQ<br>SP	###<br>###	## (##.#)<br>## (##.#)	#.## (#.##; #.##)	#.###
Neonatal anaemia	MQ<br>SP	###<br>###	## (##.#)<br>## (##.#)	#.## (#.##; #.##)	#.###
Prematurity	MQ<br>SP	###<br>###	## (##.#)<br>## (##.#)	#.## (#.##; #.##)	#.###
Placental *P. falciparum* submicroscopic infection†	MQ<br>SP	###<br>###	## (##.#)<br>## (##.#)	#.## (#.##; #.##)	#.###
Peripheral maternal submicroscopic parasitaemia at delivery†	MQ<br>SP	###<br>###	## (##.#)<br>## (##.#)	#.## (#.##; #.##)	#.###
Cord blood submicroscopic parasitaemia at delivery†	MQ<br>SP	###<br>###	## (##.#)<br>## (##.#)	#.## (#.##; #.##)	#.###
Peripheral maternal submicroscopic parasitaemia one month after delivery†	MQ<br>SP	###<br>###	## (##.#)<br>## (##.#)	#.## (#.##; #.##)	#.###

RR: Risk ratio, N: Number of subject per group, n: number of subjects in the category.
†Submicroscopic infection determined only in a subsample.

Table 13: Secondary endpoints (continous variables)

Variable	Group	N	mean	sd	Diff (95% CI)	p-value
Maternal Hemoglobin (g/dL) *when..*	MQ<br>SP	###<br>###	##.#<br>##.#	##.#<br>##.#	#.## (#.##; #.##)	#.###
Variable	Group	N	Geometric mean	IQR	PDiff (95% CI)	p-value
Gestational age (weeks) *when..*	MQ<br>SP	###<br>###	##.#<br>##.#	##.#:##.#<br>##.#: ##.#	#.## (#.##; #.##)	#.###

Diff = difference betweeng groups. PDiff= Proportional difference between group. IRQ: Interquartilic range

Table 14: Secondary endpoints (incidence)

Variable	Group	N	Events	PYAR	Incidence	RRate (95% CI)	p-value
Clinical malaria during pregnancy	MQ<br>SP	###<br>###	##<br>##	###<br>###	#.###<br>#.###	#.## (#.##; #.##)	#.###
Overall number of maternal hospital admisions	MQ<br>SP	###<br>###	##<br>##	###<br>###	#.###<br>#.###	#.## (#.##; #.##)	#.###
Outpatient number of maternal visits	MQ<br>SP	###<br>###	##<br>##	###<br>###	#.###<br>#.###	#.## (#.##; #.##)	#.###

RRatio: Relative Ratio, N: Number of subject per group, PYAR: Person years at risk.

Table 15: Secondary endpoints (endpoints for neonatal mortality)

Variable	Group	N	n(%)	RR (95% CI)	p-value
Perinatal mortality	MQ<br>SP	###<br>###	## (##.#)<br>## (##.#)	#.## (#.##; #.##)	#.###
Neonatal mortality	MQ<br>SP	###<br>###	## (##.#)<br>## (##.#)	#.## (#.##; #.##)	#.###
Early neonatal mortality	MQ<br>SP	###<br>###	## (##.#)<br>## (##.#)	#.## (#.##; #.##)	#.###

RR: Risk ratio, N: Number of subject per group, n: number of subjects in the category.
†Submicroscopic infection determined only in a subsample.

6.4 Safety (Safety, ITT)

Table 16: Summary of serious adverse events

Variable	MQ_s (N= XXX)			MQ_f (N= XXX)			SP (N= XXX)		
	n	%	(95% CI)	n	%	(95% CI)	n	%	(95% CI)
Any Serious Adverse Event	#	##.#%	(###%; ###%)	#	##.#%	(###%; ###%)	#	##.#%	(###%; ###%)
Any Serious Adverse Event related with medication	#	##.#%	(###%; ###%)	#	##.#%	(###%; ###%)	#	##.#%	(###%; ###%)
Fatal Adverse Event	#	##.#%	(###%; ###%)	#	##.#%	(###%; ###%)	#	##.#%	(###%; ###%)
Fatal Adverse Event related with medication	#	##.#%	(###%; ###%)	#	##.#%	(###%; ###%)	#	##.#%	(###%; ###%)

N= Number of subjects in the group. n= Number of subjects with at least one symptom. CI= Confidence Interval.

Table 17: Serious adverse events by MedRA term

System Organ Class (code)	Preferred Term (code)	MQ_s (N= XXX)			MQ_f (N= XXX)			SP (N= XXX)		
		n	%	(95% CI)	n	%	(95% CI)	n	%	(95% CI)
XXXXXXX XXXXXX (#######)	XXXXXX XXXXXXX (#######)	#	##.#%	(###%; ###%)	#	##.#%	(###%; ###%)	#	##.#%	(###%; ###%)
XXXXXXX XXXXXX (#######)	XXXXXX XXXXXXX (#######)	#	##.#%	(###%; ###%)	#	##.#%	(###%; ###%)	#	##.#%	(###%; ###%)

N= Number of subjects in the group. n= Number of subjects with at least one symptom. CI= Confidence Interval.

Table 18: Prevalence of adverse pregancy outcomes

Variable	MQ_s (N= XXX)			MQ_f (N= XXX)			SP (N= XXX)		
	n	%	(95% CI)	n	%	(95% CI)	n	%	(95% CI)
Prematurity	#	##.#%	(###%; ###%)	#	##.#%	(###%; ###%)	#	##.#%	(###%; ###%)
Stillbirths	#	##.#%	(###%; ###%)	#	##.#%	(###%; ###%)	#	##.#%	(###%; ###%)
Miscarriages	#	##.#%	(###%; ###%)	#	##.#%	(###%; ###%)	#	##.#%	(###%; ###%)
Congenital Malformations	#	##.#%	(###%; ###%)	#	##.#%	(###%; ###%)	#	##.#%	(###%; ###%)
Maternal death	#	##.#%	(###%; ###%)	#	##.#%	(###%; ###%)	#	##.#%	(###%; ###%)

N= Number of subjects in the group. n= Number of subjects with at least one symptom. CI= Confidence Interval.

Table 19: Adverse events

Variable	MQ_s (N= XXX) n	%	(95% CI)	MQ_f (N= XXX) n	%	(95% CI)	SP (N= XXX) n	%	(95% CI)
Any Adverse event	#	##.#%	(###%; ###%)	#	##.#%	(###%; ###%)	#	##.#%	(###%; ###%)
Any Adverse event related to medication	#	##.#%	(###%; ###%)	#	##.#%	(###%; ###%)	#	##.#%	(###%; ###%)
Any Adverse event of grade 3	#	##.#%	(###%; ###%)	#	##.#%	(###%; ###%)	#	##.#%	(###%; ###%)
Any Adverse event of grade 3 related to medication	#	##.#%	(###%; ###%)	#	##.#%	(###%; ###%)	#	##.#%	(###%; ###%)
Most frequent AE	#	##.#%	(###%; ###%)	#	##.#%	(###%; ###%)	#	##.#%	(###%; ###%)
Second frequent AE	#	##.#%	(###%; ###%)	#	##.#%	(###%; ###%)	#	##.#%	(###%; ###%)
Others AE ...	#	##.#%	(###%; ###%)	#	##.#%	(###%; ###%)	#	##.#%	(###%; ###%)

N= Number of subjects in the group. n= Number of subjects with at least one symptom. CI= Confidence Interval.

Table 20: Adverse events related to medication

Variable	MQ_s (N= XXX) n	%	(95% CI)	MQ_f (N= XXX) n	%	(95% CI)	SP (N= XXX) n	%	(95% CI)
Any Adverse event	#	##.#%	(###%; ###%)	#	##.#%	(###%; ###%)	#	##.#%	(###%; ###%)
Any Adverse event related to medication	#	##.#%	(###%; ###%)	#	##.#%	(###%; ###%)	#	##.#%	(###%; ###%)
Any Adverse event of grade 3	#	##.#%	(###%; ###%)	#	##.#%	(###%; ###%)	#	##.#%	(###%; ###%)
Any Adverse event of grade 3 related to medication	#	##.#%	(###%; ###%)	#	##.#%	(###%; ###%)	#	##.#%	(###%; ###%)
Most frequent AE	#	##.#%	(###%; ###%)	#	##.#%	(###%; ###%)	#	##.#%	(###%; ###%)
Second frequent AE	#	##.#%	(###%; ###%)	#	##.#%	(###%; ###%)	#	##.#%	(###%; ###%)
Others AE ...	#	##.#%	(###%; ###%)	#	##.#%	(###%; ###%)	#	##.#%	(###%; ###%)

N= Number of subjects in the group. n= Number of subjects with at least one symptom. CI= Confidence Interval.

6.5 Tolerability (Safety, ITT)

Table 21: Tolerability

Variable	MQ_s (N= XXX)			MQ_f (N= XXX)			SP (N= XXX)		
	n	%	(95% CI)	n	%	(95% CI)	n	%	(95% CI)
Any vomiting after dose 1 or 2	#	##.#%	(###%; ###%)	#	##.#%	(###%; ###%)	#	##.#%	(###%; ###%)
Dizziness after dose 1 or 2	#	##.#%	(###%; ###%)	#	##.#%	(###%; ###%)	#	##.#%	(###%; ###%)
At least one vomiting after dose 1	#	##.#%	(###%; ###%)	#	##.#%	(###%; ###%)	#	##.#%	(###%; ###%)
Vomiting twice or more after dose 1	#	##.#%	(###%; ###%)	#	##.#%	(###%; ###%)	#	##.#%	(###%; ###%)
Dizziness after dose 1	#	##.#%	(###%; ###%)	#	##.#%	(###%; ###%)	#	##.#%	(###%; ###%)
At least one vomiting after dose 2	#	##.#%	(###%; ###%)	#	##.#%	(###%; ###%)	#	##.#%	(###%; ###%)
Vomiting twice or more after dose 2	#	##.#%	(###%; ###%)	#	##.#%	(###%; ###%)	#	##.#%	(###%; ###%)
Dizziness after dose 2	#	##.#%	(###%; ###%)	#	##.#%	(###%; ###%)	#	##.#%	(###%; ###%)

N= Number of subjects in the group. n= Number of subjects with at least one symptom. CI= Confidence Interval.

7 Individual data listings

- List of subjects and dates of recruitment, dose1, dose2, delivery, post partum visit
- List of subjects, treatment and delivery outcome, weight, gestational age
- List of subjects and AEs
- List of subjects and SAE
- ...
