# Supplementary material for: Mortality, Morbidity, and Developmental Outcomes in Infants Born to Women Who Received Either Mefloquine or Sulfadoxine-Pyrimethamine as Intermittent Preventive Treatment of Malaria in Pregnancy: A Cohort Study
Source: PLoS Med. 2016 Feb 23;13(2):e1001964. doi: 10.1371/journal.pmed.1001964 (PMC4764647; doi:10.1371/journal.pmed.1001964)
Supplement: S5 Text — (PDF) [file pmed.1001964.s012.pdf]

Analytical Plan

For the MiPPAD Trial 1 follow-up of study infants

Author: MiPPAD Stats-WG
Version: Draft Version 2.0
Date: 05/Sep/2014
Acronym: MiPPAD (CHD1)

Contents

1. Summary of the protocol 3
   a) Introduction 3
   b) Objective and endpoints 3
   c) Study design overview 4
   d) Inclusion criteria for recruitment of MiPPAD participants 4
   e) Exclusion criteria for recruitment of MiPPAD participants 4
   f) Population of analysis 4
   g) Study sample 5
   h) Study procedures 5
3. Definitions and glossary 6
   a) Scheduled visit 6
   b) Unscheduled visit 7
   c) Household visit 7
   d) Growth Outcomes 7
   e) Psychomotor development 7
   f) Clinical malaria 8
   g) Anemia 8
   h) Hospital Admissions 9
   i) Congenital abnormalities 9
   j) Infant mortality 9
5. Database management and transformation of new variables 9
6. Statistical methods 9
7. Model tables and graphs 11
   a) Trial profile (ATP and ITT) 11
   b) Baseline characteristics 12
   c) Endpoints (ATP crude, ATP adjusted, and ITT) 12
   d) Patterns of missing data 15
8. Annexe 1 17

1. Summary of the protocol

a) Introduction

This document details the methods to be used for the processing and analysis of the data collected in the follow-up of children born during the Malaria in Pregnancy Preventive Alternative Drugs (MiPPAD) clinical trial 1, and collected under the CHD1 Case Report Forms (CRFs)

An open-label randomized three –arm clinical trial was conducted in four sub-Saharan countries (Mozambique, Benin, Gabon and Tanzania) to compare two-dose mefloquine (MQ) *versus* two-dose of sulphadoxine-pyrimethamine (SP) for intermittent preventive treatment of malaria in pregnancy (IPTp) and to compare MQ tolerability between two different MQ regimens in the context of Insecticide Treated Nets (ITNs) use. The three study arms were: 1) IPTp with SP, 2) IPTp with MQ (15 mg/kg) given as full dose on one day, 3) IPTp with MQ (15 mg/kg) split dose over two days. In total, 4749 HIV-negative women of all gravidities were enrolled at the first antenatal clinic (ANC) visit and followed until one month after the end of pregnancy. Children born from study participants were followed up until 12 months of age, through scheduled visits at month 1, 9 and 12 after birth. Outcomes on their growth, psychomotor development, morbidity and mortality will inform us on the safety of IPTp, either with MQ or SP, in the development of infants.

b) Objective and endpoints

- **<u>Objectives</u>**

➢ To assess the safety of IPTp-MQ compared to IPTp-SP, in mortality, general and malaria specific morbidity, and psychomotor development of infants born to women participating in the MiPPAD trial 1.

- **<u>Endpoints</u>**

➢ Infant mortality rate
➢ Incidence of clinical malaria
➢ Incidence of overall and malaria-specific hospital admissions
➢ Incidence of overall and malaria-specific outpatients visits
➢ Proportion of infants with:
  - Underweight, wasting and stunting at months 1, 9 and 12
  - Moderate and severe acute malnutrition at months 1, 9 and12.
  - Not achieving expected psychomotor development milestones at months 1, 9 and 12
  - Congenital abnormalities
  - Different specific -cause hospital admissions , according to MedRA Preferred Term (PT) and System Organ Class (SOC) coding system, on their first year of life

- Severe and moderate anemia (with and without associated malaria) on the first year of life
- Clinical congenital malaria

c) Study overview

It is a descriptive study using prospectively obtained data from the one-year follow up of children born to women participating in a randomized open-label trial to compare MQ with SP as IPTp in the prevention of the adverse effects of malaria during pregnancy and to compare MQ tolerability of 2 different MQ administration regimens.

The three arms of the study are:

1. IPTp with SP (SP)
2. IPTp with MQ given as full dose (MQ_f)
3. IPTp with MQ given as a split dose (MQ_s)

d) Inclusion criteria for recruitment of MiPPAD participants

1. Permanent resident in the area
2. Gestational age at the first antenatal visit ≤ 28 weeks
3. Agreement to deliver in the study site's maternity(ies) wards

e) Exclusion criteria for recruitment of MiPPAD participants

1. Residence outside the study area or planning to move out in the following 18 months from enrollment
2. Gestational age at the first antenatal visit > 28 weeks of pregnancy
3. Known history of allergy to sulfa drugs or mefloquine
4. Known history of severe renal, hepatic, psychiatric or neurological disease
5. MQ or halofantrine treatment in the preceding 4 weeks
6. HIV infection
7. Participating in other intervention studies.

f) Population of analysis

- According to protocol (ATP)

This population includes all children born to women who fulfilled all the inclusion-exclusion criteria and took the two IPTp doses, received the LLITNs and from whom data is available for the analysis.

It will be excluded from the ATP analysis if their mothers:

- Do not full fill inclusion criteria
- Have no data on birth outcomes
- Received incomplete number of IPTp administrations
- Received only half-dose of IPTp administration in the split-dose arm of MQ
- Received second IPTp half-dose administration more than 48 hours since the first one in the split arm of MQ
- Less than 4 weeks (28 days) between the two administrations of IPTp in all arms

- Received a lower dose of IPTp compared to the one specified in the protocol for the same weight.
- Received wrong study drug medication
- Birth weight recorded more than 7 days after delivery
- Did not receive a LLITN
- Had a multiple delivery

- Intention to Treat (ITT)

This population includes all children born from all randomized women. Following the intention-to-treat principle, children will be analyzed according to the preventive treatment their mothers were assigned to at the time of recruitment and randomization.

g) Study sample

For the ITT analysis we will include a sample of 4261 live births born to all trial 1 MiPPAD participants and for the ATP analysis 3348 live births born to women who fulfilled the ATP inclusion criteria.

h) Study procedures

All pregnant women attending ANC for the first time were screened for eligibility to participate in MiPPAD study. Study candidates that met inclusion criteria, accepted to participate in the study and signed informed consent were recruited, where follow-up of the women and their child was specified. They were given a study number and randomized to either SP, MQ full dose or MQ split dose group. Each participant was uniquely identified in the study by a combination of her site code and subject number. The women allocated to the SP group received 3 tablets of the fixed combination therapy containing 500 mg of sulfadoxine and 25 mg of pyrimetamine. Whereas study participants allocated to the MQ groups received 15mg/kg of MQ as IPTp. Each MQ tablet contains 250 mg of mefloquine base. The number of MQ tablets to given to the woman were administered according to the maternal weight at the time of 1st IPTp administration. Women allocated to MQ full dose group received the full IPTp dose on one time. Whereas those allocated to the MQ split dose group were administered MQ tablets in 2 days. Women were followed up according to study procedures up to one month post-partum (see MiPPAD protocol). Newborns were given a study number independent from the mother's study number in order to be uniquely identified and were followed up until 12 months of age. Mothers were asked to bring their child to the study health facilities at month 1 (or coinciding with the first EPI visit), 9 and 12 after birth for scheduled visits, were clinical and psychomotor assessment was performed and reported in specific questionnaires. In case they did not attend scheduled visits study participants were visited at home. Unscheduled outpatients visits and hospital admissions were also recorded in specific questionnaires. Procedures for each visit are specified in Table 1 and defined in section 3.

Table 1: Infants visits and procedures schedule

Study Procedure	At Birth	1 month§	9 months *	12 months *	Unscheduled visits
Medical history	X	X	X		X
Physical examination	X	X	X	X	X
Psychomotor development assessment	X	X	X	X	
MUAC		X	X	X	X
Weight	X	X	X	X	
Height	X	X	X	X	
Temperature	X	X	X	X	
Blood smear	X	+	+	+	
Haemoglobin test	X	+	+	+	

* Household visits in case they do not attend the scheduled visits at study health facilities.

+ Only if fever (≥ 37:5^{0} C) or history of fever in the past 24 hours or signs suggestive of malaria.

§ First visit will be scheduled 1 month after birth or coinciding with first EPI visit. MUAC:Middle-upper arm circumference

3. Definitions and glossary

a) Scheduled visit

At month 1 (or coinciding with 1st EPI visit), 9 and 12 after birth, study children were physically examined by study personnel. The psychomotor development of the infant was assessed following a simplified protocol described in the Appendix 1. Weight, height and axillary temperature were measured and recorded. A capillary blood sample was taken from infants with fever (axillary temperature ≥37.5ºC) or history of fever in the last 24 hours, or appearing pale, for malaria parasitemia examination and haematological determination. Scheduled visits were considered valid if they took place within one week before and four weeks after of the target date.

b) Unscheduled visit

Throughout the 1st year of follow-up, study infants reporting being sick at the health facilities were seen by study personnel. They were physically examined. Weight, height, and axillary temperature were measured and recorded. Patients with fever (axillary temperature ≥37,5ºC) or history of fever in the last 24 hours or who appear pale had a thick and thin blood film done. Blood hemoglobin was also determined.

c) Household visit

Study participants were visited at home in case they did not attend the scheduled visits at 9th and 12th month after birth.

d) Growth Outcomes

Weight (Kg) and height (cm) were determined, using monthly calibrated scales, at delivery and all study visits. According to WHO Global Database on Child Growth and Malnutrition the three recommended anthropometric indices to assess growth and nutrition on an infant study population are weight for age, weight for height and height for age. Z scores were calculated for all of them and interpreted according to WHO standard definitions (http://www.who.int/childgrowth/en/)

Stunting= height for age z-score < -2SD

Underweight= weight for age - score < -2SD

Wasting= weight for height z-score < -2SD

Severe Acute Malnutrition was defined following WHO standard definition either as:

- Weight for height z score < -3SD or
- Middle Upper Arm Circumference (MUAC) < 115mm

e) Psychomotor development

Psychomotor development of the infant was assessed by direct observation of study nurse using a simplified protocol adapted from the *Anamnesis and Physical Examination, Lissauer T* (see appendix 1). This simplified protocol included the verification of several development milestones the child is expected to achieve at the different time points. Children´s performance will be analyzed according to the differences in the proportions of not achieved psychomotor development milestones for each month.

Psychomotor development by month 1

- Proportion of infants who do not move the four extremities symmetrically by month 1

- Proportion of infants with abnormal muscle tone by month 1
- Proportion of infants unable to follow objects by month 1
- Proportion of infants who do not respond to sounds by month 1
- Proportion of infants who do not respond to smiles by month 1

Psychomotor development by month 9

- Proportion of infants who are not able to seat without leaning by month 9
- Proportion of infants who are unable to crawl by month 9
- Proportion of infants who are not able to stand without help by month 9
- Proportion of infants who are not able to walk without support by month 9
- Proportion of infants who are not able to do palm grasp by month 9
- Proportion of infants who are not able to move the objects from one hand to the other by month 9
- Proportion of infants not turning at someone's voice by month 9
- Proportion of infants who are not able to say any word by month 9
- Proportion of infants who are not able to bring solid food to his/her mouth

Psychomotor development by month 12

- Proportion of infants who are not able to walk by month 12
- Proportion of infants who are not able to do pincer grasping by month 12
- Proportion of infants who are not able to understand orders by month 12
- Proportion of infants who are not able to say some words by month 12
- Proportion of infants who are not able to drink from a cup by month 12

f) Clinical malaria

Clinical malaria is defined as fever (≥37, 5º C) or history of fever in the past 24 hours or signs suggestive of malaria, through direct questioning on scheduled visits and active reporting in unscheduled visits, confirmed by a positive blood smear.

g) Clinical congenital malaria

Presence of asexual *Plasmodium* parasites of any species in cord blood or in the newborn peripheral blood within the first 7 days of life (or later if there is no possibility of a postpartum infection by a mosquito bite, e.g. being out in an endemic area) with any of the following: fever, anemia, respiratory distress, hemodynamic shock, jaundice, hepato or spleno- megaly.

h) Anemia

Anemia is defined as a haemoglobin (Hb) concentration <13g/dl in peripheral blood and Hb< 12.5 g/dl in cord blood for neonates and Hb < 11 g/dl in infants , measured by HemoCue rapid test in scheduled and unscheduled visits whenever malaria was suspected.

i) Hospital Admissions

Hospital admission of children born to MiPPAD study participants were considered to be a Serious Adverse Event (SAE) and were supervised and reported in specific questionnaires by study clinician and notified to the sponsor (CRESIB) and the Data Safety Monitoring Board (DSMB). Diagnosis for the SAE reporting were codified and classified using MedRA Preferred Term (PT) and System Organ Class (SOC) coding system.

j) Congenital abnormalities

Congenital abnormalities were considered to be a Serious Adverse Event (SAE) and had to be reported in a specific questionnaire and notified to the sponsor (CRESIB) and the Data Safety Monitoring Board (DSMB). They were assessed at birth by study nurse and confirmed by study clinician. Assessment of any congenital abnormalities was performed in all scheduled visits.

k) Infant mortality

Deaths were considered to be a Serious Adverse Event (SAE) and had to be reported in a specific questionnaire and notified to the sponsor (CRESIB) and the Data Safety Monitoring Board (DSMB). As the minimum, all the study mothers and her children who did not attend month 12 scheduled visit were visited at home for final assessments of status.

5. Database management and transformation of new variables

A centralized data management and data cleaning was conducted at the site in Manhiça (Mozambique) with the lead of a highly trained statistician. The data management team, with collaboration with all five sites, developed case report forms (CRFs) and provided training to all sites on data collection methodology, data quality control (QC), and quality assurance (QA). Each CRF was identified by a unique serial number. These CRFs were entered, following standard procedures, into OpenClinica 3.1. OpenClinica has a dataset extraction function which allows each CRF to be extracted as one dataset in multiple formats (eg: SPSS, csv, postgresdatamart etc and converted into stata. Subsequently, the entered data was systematically checked by Data Management staff, using error messages printed from validation programs and database listings. Errors on data entry not reflected on CRF were corrected by Data Management personnel. Other errors or omissions were entered on Data Query Forms, which were returned to the study site for resolution. The signed original and resolved Data Query Forms were kept with the CRFs at the study site, and a copy was sent to the CISM so the resolutions can be entered into the database. Quality control audits of all key safety and efficacy data in the database were made prior to locking the database.

6. Statistical methods

Baseline characteristics of patients at delivery will be described using standard statistics. Continuous variables will be summarized by sample size, mean, median, standard deviation. Discrete variables will be summarized by frequencies and percentages.

Proportions will be compared between IPTp groups using Fisher's exact test for covariates and possible confounder are done using binomial regression with log link, and using robust estimates of the covariance (huber method) using the method proposed by Zou (Zou G., A modi_ed poisson regression approach to prospective studies with binary data. American Journal of Epidemiology. 2004;159(7):702-706). They will presented as a Risk Ratio (RR) or a reduction in the RR (1 - RR * 100%) if the RR is lower than 1. Continuous variables will be compared between groups and adjusted for covariates and possible co- founders, such as baseline characteristics at delivery, seasonality or country, using ordinary least square regression. Variables will be transformed to the logarithm scale if normality is improved and result presented as Proportional Difference.

Z-scores for nutritional status evaluation (weight for height, height for age and weight for age) were calculated using the WHO Child Growth Standard 2006 (World Health Organization: WHO child growth standards: length/height-for-age, weight-for-age, weight-for-length, weight-for-height and body mass index-for-age: methods and development. *Geneva: World Health Organization* 2006:312.) with the command "zanthro" in Stata (Vidmar S, Carlin J, Hesketh K, Cole T: Standardizing anthropometric measures in children and adolescents with new functions for egen. *Stata Journal* 2004, 4:50–55). Patterns of missing values for growth outcomes are shown on table 11.

Incidence of clinical malaria, malaria -related anemia, infant mortality, overall admissions and outpatient attendances will be estimated as the number of episodes over the time at risk. Time at risk is estimated as the time from the start of follow up (day of birth in infants) until the end of follow-up (visit after 12 months of age in infants) or withdrawal due to censoring or death, whatever occurs first. In order to avoid counting twice the same episode of clinical malaria and malaria- related to anemia, subjects will not contribute to the denominator or numerator during an arbitrary period of 28 days after an event of clinical malaria confirmed. For admissions and outpatient attendances a maximum of one episode per day will be allowed. The total number of events will be compared between groups using negative binomial regression to take into account a possible extra Poisson variation due to different frailty of the subjects. The comparison will be expressed as Relative Rate (RRate)

Data analysis will be performed using Stata (Stata Corporation, College Station, TX, USA).

7. Model tables and graphs

a) Trial profile (ATP and ITT)

Table 1: MiPPAD trial 1 children's trial profile

**Mother´s IPTp group**	**MQ_s**	**MQ_f**	**SP**
Live births (N)	#	#	#
Visit 1 month after delivery (n, %)	#(#.#%)	#(#.#%)	#(#.#%)
Visit 9 month after delivery (n, %)	#(#.#%)	#(#.#%)	#(#.#%)
Visit 12 month after delivery (n, %)	#(#.#%)	#(#.#%)	#(#.#%)

Tables 2, 3 and 4: Reasons for children´s withdrawal before visit 1, 2 and 3

**Mother´s IPTp group**	**MQ_s**	**MQ_f**	**SP**
Migration (n, %)	#(#.#%)	#(#.#%)	#(#.#%)
Not found (n, %)	#(#.#%)	#(#.#%)	#(#.#%)
Absent (n, %)	#(#.#%)	#(#.#%)	#(#.#%)
Refusal (n, %)	#(#.#%)	#(#.#%)	#(#.#%)
Death (n, %)	#(#.#%)	#(#.#%)	#(#.#%)
Other (n, %)	#(#.#%)	#(#.#%)	#(#.#%)

Table 5: Reasons for children´s withdrawal before completion of follow up

**Mother´s IPTp group**	**MQ_s**	**MQ_f**	**SP**
Death (n, %)	#(#.#%)	#(#.#%)	#(#.#%)
Serious Adverse Event (n, %)	#(#.#%)	#(#.#%)	#(#.#%)
Consent withdrawal (n, %)	#(#.#%)	#(#.#%)	#(#.#%)
Migration (n, %)	#(#.#%)	#(#.#%)	#(#.#%)
Lost to follow up (n, %)	#(#.#%)	#(#.#%)	#(#.#%)
Other (n, %)	#(#.#%)	#(#.#%)	#(#.#%)

b) Baseline characteristics

Table 6: Baseline characteristics at birth

Mothers IPTp group		MQ_s[1]	MQ_f[2]	SP[3]
Live births by country (n(%))	Benin	#(#.#%)	#(#.#%)	#(#.#%)
	Gabon	#(#.#%)	#(#.#%)	#(#.#%)
	Mozambique	#(#.#%)	#(#.#%)	#(#.#%)
	Tanzania	#(#.#%)	#(#.#%)	#(#.#%)
Sex				
- Males (n (%))		#(#.#%)	#(#.#%)	#(#.#%)
- Females (n (%))		#(#.#%)	#(#.#%)	#(#.#%)
Weight (g, mean(sd))		#(#.#)	#(#.#)	#(#.#)
Length (cm, mean(sd))		#(#.#)	#(#.#)	#(#.#)
Head circumference (cm, mean (sd))		#(#.#)	#(#.#)	#(#.#)
Cord blood anemia (n (%))		#(#.#)	#(#.#)	#(#.#)
Cord blood parasitemia (n (%))		#(#.#)	#(#.#)	#(#.#)
Gestational age (weeks, median(IQR))		#(#.#)	#(#.#)	#(#.#)
Prematurity (n (%))[4]		#(#.#)	#(#.#)	#(#.#)
Low birth weight (n (%))[4]		#(#.#)	#(#.#)	#(#.#)
Congenital abnormalities (n (%))		#(#.#)	#(#.#)	#(#.#)

N: Number of subjects in the group, sd: Standard deviation, n: Number of subjects in the category IQR: Interquartile range

[1] Mefloquine split-dose [2] Mefloquine full-dose [3] Sulphadoxine-Phyrimetamine [4] Data only available from Mozambique, Benin and Gabon

c) Endpoints (ATP crude, ATP adjusted, and ITT)

Tables 7: Psychomotor development outcomes: impaired psychomotor development by study group at month 1, 9 and 12

Variable	Group	N	n (%)	RR (95% CI)	p-value
**Visit month 1**					
Infants who do not move the four extremities symmetrically	MQ<br>SP	###<br>###	## (##.#)<br>## (##.#)	#.## (#.##; #.##)	#.###
Infants with abnormal muscle tone	MQ<br>SP	###<br>###	## (##.#)<br>## (##.#)	#.## (#.##; #.##)	#.###
Infants unable to follow objects	MQ<br>SP	###<br>###	## (##.#)<br>## (##.#)	#.## (#.##; #.##)	#.###

Infants who do not respond to sounds	MQ<br>SP	###<br>###	## (##.#)<br>## (##.#)	#.## (#.##; #.##)	#.###
Infants who do not respond to smiles	MQ<br>SP	###<br>###	## (##.#)<br>## (##.#)	#.## (#.##; #.##)	#.###
**Visit month 9**					
Infants who are not able to seat without leaning	MQ<br>SP	###<br>###	*## (##.#)*<br>*## (##.#)*	*#.## (#.##; #.##)*	#.###
Infants who are unable to crawl	MQ<br>SP	###<br>###	## (##.#)<br>## (##.#)	#.## (#.##; #.##)	#.###
Infants who are unable to stand without help	MQ<br>SP	###<br>###	## (##.#)<br>## (##.#)	#.## (#.##; #.##)	#.###
Infants who are unable to walk without support	MQ<br>SP	###<br>###	## (##.#)<br>## (##.#)	#.## (#.##; #.##)	#.###
Infants who are unable to do palm grasp	MQ<br>SP	###<br>###	## (##.#)<br>## (##.#)	#.## (#.##; #.##)	#.###
Infants who are unable to move the objects from one hand to the other	MQ<br>SP	###<br>###	## (##.#)<br>## (##.#)	#.## (#.##; #.##)	#.###
Infants not turning at someone's voice	MQ<br>SP	###<br>###	## (##.#)<br>## (##.#)	#.## (#.##; #.##)	#.###
Infants who are unable to say any word	MQ<br>SP	###<br>###	## (##.#)<br>## (##.#)	#.## (#.##; #.##)	#.###
Infants who are unable to bring solid food to his/her mouth	MQ<br>SP	###<br>###	## (##.#)<br>## (##.#)	#.## (#.##; #.##)	#.###
**Visit month 12**					
Infants who are unable to walk	MQ<br>SP	###<br>###	## (##.#)<br>## (##.#)	#.## (#.##; #.##)	#.###
Infants who are not able to do pincer grasping	MQ<br>SP	###<br>###	## (##.#)<br>## (##.#)	#.## (#.##; #.##)	#.###
Infants who are unable to understand orders	MQ<br>SP	###<br>###	## (##.#)<br>## (##.#)	#.## (#.##; #.##)	#.###
Infants who are unable to say some words	MQ<br>SP	###<br>###	## (##.#)<br>## (##.#)	#.## (#.##; #.##)	#.###
Infants who are unable to drink from a cup	MQ<br>SP	###<br>###	## (##.#)<br>## (##.#)	#.## (#.##; #.##)	#.###

RR: Risk ratio, N: Number of subject per group attending the scheduled visit, n: number of subjects in the category.

Table 8: Growth outcomes: stunting, underweight, wasting and acute severe malnutrition at month 1, 9 and 12

Variable	Group	N	n (%)	RR (95% CI)	p-value
**Visit month 1**					
Stunting<br>(height for age < -2SD)	MQ<br>SP	###<br>###	## (##.#)<br>## (##.#)	#.## (#.##, #.##)	#.###
Underweight<br>(weight for age < -2SD)	MQ<br>SP	###<br>###	## (##.#)<br>## (##.#)	#.## (#.##, #.##)	#.###
Wasting<br>(weight for height < -2SD)	MQ<br>SP	###<br>###	## (##.#)<br>## (##.#)	#.## (#.##, #.##)	#.###
Severe Acute Malnutrition					

-weight for height < -3SD<br><br>-MUAC<115cm)	MQ<br>SP<br>MQ<br>SP	###<br>###<br>###<br>###	## (##.#)<br>## (##.#)<br>## (##.#)<br>## (##.#)	#.## (#.##, #.##)<br><br>#.## (#.##, #.##)	#.###<br><br>#.###
**Visit month 9**					
Stunting<br>(height for age < -2SD)	MQ<br>SP	###<br>###	## (##.#)<br>## (##.#)	#.## (#.##, #.##)	#.###
Underweight<br>(weight for age < -2SD)	MQ<br>SP	###<br>###	## (##.#)<br>## (##.#)	#.## (#.##, #.##)	#.###
Wasting<br>(weight for height < -2SD)	MQ<br>SP	###<br>###	## (##.#)<br>## (##.#)	#.## (#.##, #.##)	#.###
Severe Acute Malnutrition<br>-weight for height < -3SD<br><br>-MUAC<115cm)	 MQ<br>SP<br>MQ<br>SP	 ###<br>###<br>###<br>###	 ## (##.#)<br>## (##.#)<br>## (##.#)<br>## (##.#)	 #.## (#.##, #.##)<br><br>#.## (#.##, #.##)	 #.###<br><br>#.###
**Visit month 12**					
Stunting<br>(height for age < -2SD)	MQ<br>SP	###<br>###	## (##.#)<br>## (##.#)	#.## (#.##, #.##)	#.###
Underweight<br>(weight for age < -2SD)	MQ<br>SP	###<br>###	## (##.#)<br>## (##.#)	#.## (#.##, #.##)	#.###
Wasting<br>(weight for height < -2SD)	MQ<br>SP	###<br>###	## (##.#)<br>## (##.#)	#.## (#.##, #.##)	#.###
Severe Acute Malnutrition<br>-weight for height < -3SD<br><br>-MUAC<115cm)	 MQ<br>SP<br>MQ<br>SP	 ###<br>###<br>###<br>###	 ## (##.#)<br>## (##.#)<br>## (##.#)<br>## (##.#)	 #.## (#.##, #.##)<br><br>#.## (#.##, #.##)	 #.###<br><br>#.###

RR: Risk ratio, N: Number of subject per group attending the scheduled visit, n: number of subjects in the category.

Table 9: Morbidity and mortality outcomes: incidence of hospital admissions, outpatient visits, clinical malaria and anemia and mortality on 1st year of life

Variable	Group	N	PYAR	Incidence	RR (95% CI)	p-value
All cause hospital admissions	MQ<br>SP	###<br>###	###<br>###	#.###<br>#.###	#.## (#.##, #.##)	#.###
All cause outpatient visits	MQ<br>SP	###<br>###	###<br>###	#.###<br>#.###	#.## (#.##, #.##)	#.###
Clinical malaria	MQ<br>SP	###<br>###	###<br>###	#.###<br>#.###	#.## (#.##, #.##)	#.###
Anemia in children with malaria episodes	MQ<br>SP	###<br>###	###<br>###	#.###<br>#.###	#.## (#.##, #.##)	#.###
Infant mortality	MQ<br>SP	###<br>###	###<br>###	#.###<br>#.###	#.## (#.##, #.##)	#.###

RR: Relative Rate, N: Number of subjects per group, PYAR: Person Years at risk

Table 10: Children hospital admissions classified by MedRA term

System Organ Class (code)	Preferred Term (code)	MQ_S			MQ_f			SP		
		n	%	95%CI	n	%	95%CI	n	%	95%CI
XXXXXXX (######)	XXXXXXX(######)	###	#.###	(#.##, #.##)	###	#.###	(#.##, #.##)	###	#.###	(#.##, #.##)
XXXXXXX (######)	XXXXXXX (######)	###	#.###	(#.##, #.##)	###	#.###	(#.##, #.##)	###	#.###	(#.##, #.##)
XXXXXXX (######)	XXXXXXX (######)	###	#.###	(#.##, #.##)	###	#.###	(#.##, #.##)	###	#.###	(#.##, #.##)

N=Number of subjects with at least one syptom, CI=Confidence Interval

d) Patterns of missing data

Table 11: Patterns of missing values for growth outcomes for visit 1, 2 and 3

Visit	Growth Z-scores					Growth variables		
	WAZ[1]	WHZ[2]	HAZ[3]	n/N[4]	%	Height	Weight	Age
Month	X	.	.			X	.	.
						X	X	.
						X	.	X
						.	.	X
						.	X	.
						.	X	X
	X	X	.			X	.	.
						X	X	.
						X	.	X
						.	.	X
						.	X	.
						.	X	X
	X	.	X			X	.	.
						X	X	.
						X	.	X
						.	.	X
						.	X	.
						.	X	X
	.	.	X			X	.	.
						X	X	.
						X	.	X
						.	.	X
						.	X	.
						.	X	X
	.	X	.			X	.	.
						X	X	.
						X	.	X
						.	.	X
						.	X	.
						.	X	X
	.	X	X			X	.	.
						X	X	.
						X	.	X

						.	.	X
						.	X	.
						.	X	X

[1] Weight for age Z-score [2] Weight for height Z-score [3]Height for age Z-score [4] Number of missing values out of the total number of visits

8. Annexe 1

<u>Appendix 5. Psychomotor Development test</u>

1 Newborn: assessments at birth

a) Figure 1

	Expected findings include:
Gross motor skills	Symmetric and anti-gravitational movements of the 4 extremities (figures 2 and 3). Normal muscle tone
Language	Crying
Social skills	Response to pinch
Audition	Quiet with the voice; frighten by great noises
Vision	Looks at the face; responses to light

b) Figure 2

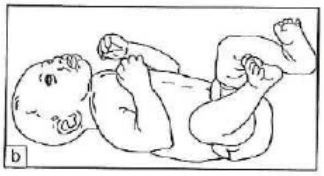

Dorsal decubitus:
Symmetric position, bended extremities

c) Figure 3

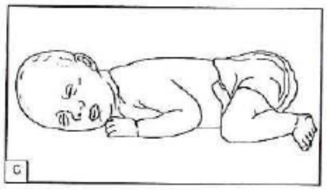

Ventral decubitus:
Flexion position, bended knees

2 Infants 6-8 weeks of age

a) Figure 1

	Expected findings include:
Gross motor skills	Symmetric movements of the 4 extremities (figures 2 and 3). Normal muscle tone
Fine motor skills	Eyes follow objects (6 weeks) (figure 4); conjugated movement is observed; no strabismus or nistagmus
Language/Audition	Normal crying; responses to sounds
Social skills	Response to smiles (figure 4)
Physical examination	Weight, height, to rule out congenital malformations

b) Figure 2

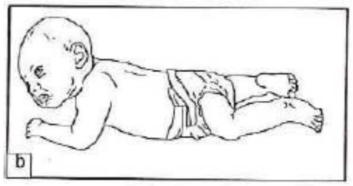

Ventral decubitus:
The head stands at 45º

c) Figure 3

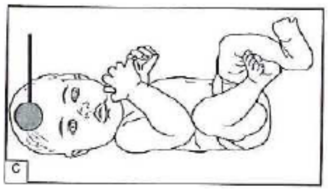

Dorsal decubitus:
Follows an object in movement turning the head

d) Figure 4

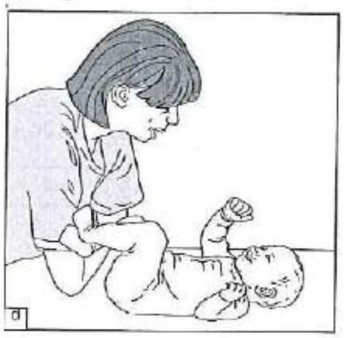

Smiles at some stimuli

3 Assessments at 6-9 months of age

a) Figure 1

	Expected findings include:
Gross motor skills	Seats without leaning (six months) (figure 2)<br>Can stand up (seven months)<br>Tractions to stand up (nine months)
Fine motor skills /vision	Tries to catch small objects, holds them by palm grasp (figure 3) and passes them from one hand to the other (six months)<br>Can bang objects holding them in different hands<br>No strabismus
Language/Audition	Turns at someone's voice (seven months)<br>Babbles ; says "daddy", "mammy" (ten months) at random
Social skills	Can bring solid food to his/her mouth (six months)
Physical examination	Weight, height

b) Figure 2

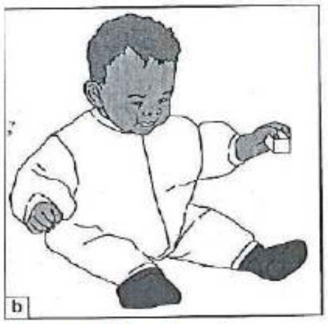

Seats without leaning

c) Figure 3

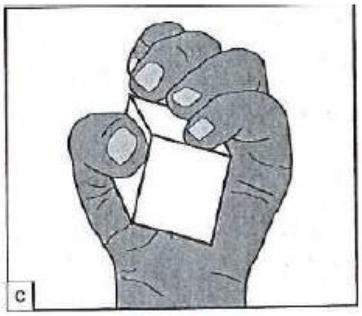

Palm grasp

4 Assessments at 12 months of age

a) Figure 1

	Expected findings include:
Gross motor skills	Walks leaning on furniture (nine months)<br>Can walk with help (figure 2) and does some steps without leaning (twelve months)
Fine motor skills /vision	Efficacious pincer grasping between the thumb and forefinger (figure 3) of small objects (10,5 months)<br>No observed strabismus, neither according to parents
Language/Audition	Understands orders like "no", "bring"; correctly says "daddy", "mammy" and a few more words (thirteeen months)
Social skills	Drinks from a glass (twelve months)<br>Eats with his/her hands or with a spoon<br>Says "goodbye" with his/her hand<br>Social response: is scared of strangers

b) Figure 2

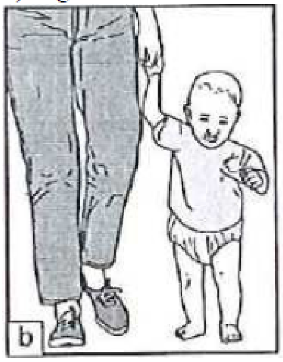

Walks with help

c) Figure 3

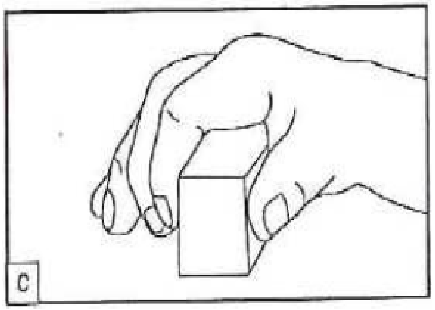

Pincer grasp

....

Adapted from Lissauer T. Anamnesis and physical examination. In: Lissauer T, Clayden G. Illustrated textbook of paediatrics. First edition. Mosby-Year Book. 1999.
