# Supplementary material for: Mortality, Morbidity, and Developmental Outcomes in Infants Born to Women Who Received Either Mefloquine or Sulfadoxine-Pyrimethamine as Intermittent Preventive Treatment of Malaria in Pregnancy: A Cohort Study
Source: PLoS Med. 2016 Feb 23;13(2):e1001964. doi: 10.1371/journal.pmed.1001964 (PMC4764647; doi:10.1371/journal.pmed.1001964)
Supplement: S6 Text — (PDF) [file pmed.1001964.s013.pdf]

Corporació Sanitària

CLÍNIC BARCELONA Hospital Universitari

Dña. Begoña Gómez Pérez, Secretaria del Comité Ético de Investigación Clínica del Hospital Clínic i Provincial de Barcelona,

CERTIFICA:

Que este Comité, con fecha 09/10/08, ha evaluado la propuesta del promotor para que se realice el ensayo clínico código de protocolo IP.07.31080.002 **titulado "Evaluation of the safety and efficacy of mefloquine as intermittent preventive treatment in pregnacy". Versión 01 de 25/09/08; CI: versión de 25/09/08**, y considera que:

. Se cumplen los requisitos necesarios de idoneidad del protocolo en relación con los objetivos del estudio y están justificados los riesgos y molestias previsibles para el sujeto.

. La capacidad del investigador y los medios disponibles son apropiados para llevar a cabo el estudio.

. Son adecuados tanto el procedimiento para obtener el consentimiento informado como la compensación prevista para los sujetos por daños que pudieran derivarse de su participación en el ensayo.

. El alcance de las compensaciones económicas previstas no interfiere con el respeto a los postulados éticos.

Que este Comité acepta que dicho ensayo clínico sea realizado en el Centro de Investigaçao em Saúde de Manhiça (CISM), Mozambique por la **Dra. Menéndez Santos, Clara** como investigador principal, debiendo ser comunicado a dicho Comité Ético todo cambio en el protocolo o acontecimiento adverso grave.

Lo que firmo en Barcelona, a 17 de octubre de 2008

CLÍNIC BARCELONA Hospital Universitari
COMITÈ ÈTIC INVESTIGACIÓ CLÍNICA

CIF - G-08431173

HOSPITAL CLÍNIC I PROVINCIAL DE BARCELONA
Villarroel, 170 - 08036 Barcelona (España)
Tel. 93 227 54 00 Fax 93 227 54 54
www.hospitalclinic.org

852
